# Nanomedicine Penetrating Blood‐Pancreas Barrier for Effective Treatment of Acute Pancreatitis

**DOI:** 10.1002/advs.202413925

**Published:** 2025-02-14

**Authors:** Dan Wang, Shuya Wang, Jinjin Liu, Xiaojing Shi, Tingli Xiong, Ruishi Li, Wei Wei, Liandong Ji, Qiong Huang, Xuejun Gong, Kelong Ai

**Affiliations:** ^1^ Department of General Surgery Xiangya Hospital Central South University Changsha 410008 China; ^2^ Xiangya School of Pharmaceutical Sciences Central South University Changsha 410013 China; ^3^ Department of Pharmacy Xiangya Hospital Central South University Changsha 410008 China; ^4^ National Clinical Research Center for Geriatric Disorders Xiangya Hospital Central South University Changsha 410008 China; ^5^ Hunan Provincial Key Laboratory of Cardiovascular Research Xiangya School of Pharmaceutical Sciences Central South University Changsha 410013 China; ^6^ Key Laboratory of Aging‐related Bone and Joint Diseases Prevention and Treatment Ministry of Education Xiangya Hospital Central South University Changsha 410008 China

**Keywords:** acute pancreatitis, blood‐pancreas barriers, cGAS, mitochondrial targeting, reactive oxygen species, STING

## Abstract

Acute pancreatitis (AP) is a primary contributor to hospitalization and in‐hospital mortality worldwide. Targeted elimination of mitochondrial reactive oxygen species (mtROS) within pancreatic acinar cells (PACs) represents an ideal strategy for treating AP. However, existing drugs fail to overcome the physiological barriers of the pancreas to effectively reach PACs mitochondria due to the trade‐off between conventional positively charged mitochondrial‐targeting groups and their inability to penetrate the blood‐pancreas barrier (BPB). Here, a tungsten‐based heteropolyacid nano‐antioxidant (mTWNDs) is introduced, co‐modified with tannic acid (TA) and melanin, enabling site‐specific clearance of mtROS in PACs, offering a highly effective treatment for AP. TA exhibits a strong affinity for proline‐rich type III collagen and the mitochondrial outer membrane protein TOM20. This unique property allows mTWNDs to traverse the damaged BPB‐exposing type III collagen to reach PACs and subsequently penetrate mitochondria for targeted mtROS elimination. In cerulein‐induced AP mice, mTWNDs reversed AP at 1/50th the dose of N‐acetylcysteine, suppressing PACs apoptosis and inflammation by blocking the stimulator of the interferon genes pathway activation in macrophage. This study establishes a mitochondrial‐targeting antioxidant nanomedicine strategy for AP treatment.

## Graphical abstract

This study introduces tungsten‐based heteropolyacid nano‐antioxidants (mTWNDs), which are co‐modified with tannic acid (TA) and melanin (A). It is able to cross the damaged blood‐pancreas barrier (BPB) to reach the mitochondria of the pancreatic acinar cells (PACs) and specifically scavenge reactive oxygen species (ROS)  to treat acute pancreatitis (AP) (B‐C). Mechanistically, mTWNDs inhibit the release of mitochondrial DNA (mt‐DNA) by suppressing apoptosis of PACs, thereby attenuating the stimulator of the interferon genes (STING)‐induced inflammation (D).

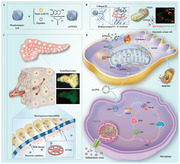

## 1 Introduction

Acute pancreatitis (AP), featuring autodigestion, edema, and hemorrhage of pancreatic tissue, is a common gastrointestinal emergency. The worldwide incidence of AP is increasing at an annual rate of ≈3.07%.^[^^]^ It ranks as the second leading reason for hospitalization among gastrointestinal disorders and significantly contributes to high healthcare costs, imposing a substantial burden on healthcare systems worldwide.^[^ ^3^ ^]^ Moreover, about one‐fifth of cases of AP progress to moderate or severe AP, which is associated with local or systemic complications, carrying a mortality rate of ≈30%.^[^ ^4^ ^]^ Current treatments are limited to fluid resuscitation and supportive care, as no precise targeted therapies for AP have been developed.^[^ ^5^ ^]^ A major challenge in advancing treatment is the natural structure of the blood‐pancreas barrier (BPB), which severely restricts the penetration of conventional medicines into pancreatic tissue.^[^ ^6^ ^]^ Thus, developing precise treatment strategies for AP remains a critical and urgent need.

Although numerous factors can contribute to AP, the over‐generated mitochondrial reactive oxygen species (mtROS) is a pivotal factor in its complex pathogenesis.^[^ ^7^ ^]^ Excessive mtROS leads directly to mitochondrial breakage and triggers cytochrome c (Cyt c)‐mediated apoptosis of pancreatic acinar cells (PACs).^[^ ^8^ ^]^ Furthermore, macrophage activation during AP is strongly associated with the overproduction of reactive oxygen species (ROS).^[^ ^9^ ^]^ Macrophages hold a central position in the AP inflammatory response. Elevated mtROS levels lead to the leakage of mitochondrial DNA (mt‐DNA) from PACs, which binds to cyclic GMP‐AMP synthase (cGAS) in macrophages. This recognition initiates the downstream stimulator of the interferon genes (STING) pathway, leading to the secretion of pro‐inflammatory factors and amplifying the inflammatory response.^[^ ^10^, ^11^ ^]^ These inflammatory mediators further exacerbate mtROS generation, perpetuating a vicious cycle.^[^ ^12^, ^13^ ^]^ Consequently, targeting mtROS bursts presents a promising therapeutic approach for AP.

To successfully address mtROS bursts in PACs, antioxidants must overcome two major barriers: penetration of the BPB to reach PACs and subsequent delivery to PACs mitochondria. To date, no antioxidant has been identified that can overcome both these barriers to effectively treat AP. The BPB, akin to the blood‐brain barrier, separates pancreatic tissue from the bloodstream and comprises a capillary endothelial cell layer, a basement membrane, and PACs. This barrier selectively filters and limits the permeability of drugs based on molecular size and structure.^[^ ^6^ ^]^ The capillaries within the BPB are enriched with a dense type III collagen matrix, which is further shielded by a glycocalyx layer on its surface.^[^ ^14^ ^]^ Various inflammatory factors and matrix metalloproteinases produced during AP can damage the dense glycocalyx structure, exposing the type III collagen and compromising the BPB.^[^ ^15^ ^]^ Additionally, mitochondrial targeting moieties typically consist of molecules with a strong affinity for mitochondrial outer membrane proteins. However, these molecules are often highly positively charged, rendering drugs modified with these moieties unable to recognize and traverse the damaged BPB.^[^ ^16^ ^]^ A review of the literature indicates that both the mitochondrial outer membrane protein TOM20 and type III collagen are abundant in proline residues. Tannic acid (TA), a plant‐derived polyphenol, has demonstrated a strong binding capacity for proline‐rich mucins.^[^ ^17^ ^]^ Based on this evidence, we hypothesize that TA‐modified nanomedicines could enable an active targeting strategy, facilitating drug delivery from the impaired BPB to mitochondria in PACs.

**Scheme 1:**
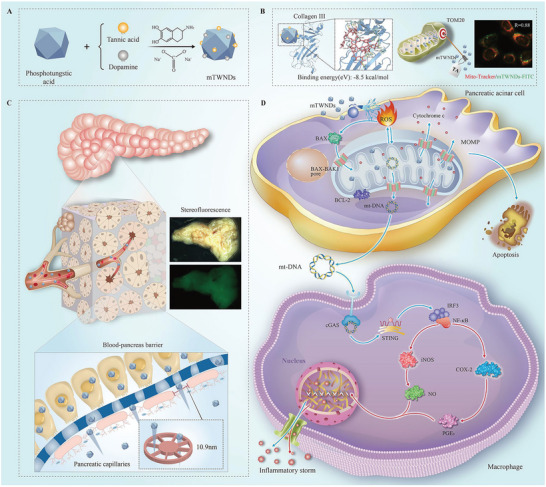
Schematic mechanism of mTWNDs for AP and characteristics of mTWNDs. A) The mTWNDs were synthesized by using TA and dopamine in an alkaline environment, which allows the reduction of phosphotungstic acid. B) The mTWNDs exhibit a high affinity for type III collagen and TOM20, enabling efficient recognition of the damaged BPB and subsequent targeting of PACs mitochondria. C) The ultrasmall particle size facilitates mTWNDs penetration through the impaired BPB, delivering the nanomedicines to the pancreas. D) The therapeutic mechanisms of mTWNDs include scavenging ROS, inhibiting apoptosis, blocking the cGAS/STING pathway, and suppressing inflammatory factors released by macrophages.

Here, we developed TA‐modified ultrasmall nanomedicines (mTWNDs) with potent antioxidant activity to specifically eliminate mtROS in PACs. The mTWNDs were synthesized by reducing tungsten‐based heteropolyacids with TA and dopamine under alkaline conditions (**Scheme** **1**A). During synthesis, W^6+^ in the heteropolyacid is reduced to W^5+^, while dopamine undergoes oxidation and polymerization to form melanin, also known as polydopamine (PDA). The resulting mTWNDs exhibit a high W^5+^/W^6+^ ratio, enabling efficient scavenging of O_2_^•−^, ·OH, H_2_O_2_, and ONOO^−^. In a cerulein‐induced AP mouse model, mTWNDs demonstrated exceptional therapeutic efficacy, achieving results comparable to conventional antioxidant drug N‐acetylcysteine (NAC) at just 1/50th the dosage. The superior therapeutic effects of mTWNDs can be attributed to several mechanisms. First, TA modification imparts a high affinity for type III collagen and TOM20, allowing mTWNDs to efficiently target the damaged BPB and subsequently localize to PACs mitochondria (Scheme 1B). Second, the ultrasmall particle size of mTWNDs facilitates penetration of the compromised BPB, enabling delivery to the pancreas (Scheme 1C). Finally, the PDA‐modified mTWNDs effectively scavenge mtROS, reducing PACs apoptosis and protecting mitochondria. This mitochondrial protection limits mt‐DNA release, thereby inhibiting cGAS/STING‐mediated inflammation in macrophages (Scheme 1D).

## 2 Results

### 2.1 Preparation and Characterization of mTWNDs

**Figure 1:**
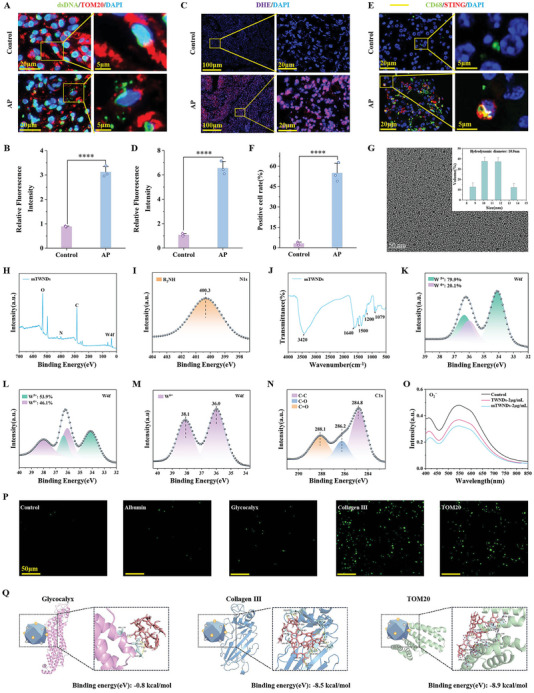
Characterization of mTWNDs. A) Immunofluorescent labeling of dsDNA, TOM20, and DAPI in human normal and pancreatitis pancreatic samples. B) Quantitative analysis of cytoplasmic dsDNA from panel (A). C) DHE and DAPI staining of human normal and pancreatitis pancreatic tissues. D) Quantitative analysis of DHE staining from panel (C). E) Immunofluorescence staining of CD68 and STING in pancreatic tissues. F) Quantitative analysis of CD68 and STING immunofluorescence from panel (E). G) TEM visualization of mTWNDs (Inset: hydrodynamic diameter distribution of mTWNDs). Scale bar: 50 nm. H) Total spectral energy of mTWNDs. I) N1s narrow‐scan XPS spectrum of mTWNDs. J) FTIR spectrum of mTWNDs. K) W4f narrow‐scan XPS spectrum of mTWNDs. L) W4f narrow‐scan XPS spectrum of TWNDs. M) XPS scan (W4f narrow) of the reaction of mTWNDs with H_2_O_2_. N) XPS scan (C1s narrow) of the reaction of mTWNDs with H_2_O_2_. O) O_2_^•−^ scavenging ability of mTWNDs and TWNDs. P) Protein‐coated plate experiment with scale bar: 50 µm. Q) Molecular docking analysis of TA with glycogen, collagen III, and TOM20 proteins. Data were displayed as mean ± standard deviation (S.D.), derived from three independent experiments (*n =* 3). The unpaired t‐tests were utilized for statistical analysis. *P*‐values were expressed as follows: ^*^ for *p <* 0.05, ^**^ for *p <* 0.01, ^***^ for *p <* 0.001, ^****^ for *p <* 0.0001 versus the control group.

To investigate the pathophysiological features of AP, we analyzed normal and AP pancreatic tissues using histological methods. Immunofluorescence analysis of double‐stranded DNA (dsDNA) and TOM20 revealed significant mt‐DNA leakage from PACs in AP tissues, which was absent in normal pancreatic tissue (**Figure** **1**A,B). Furthermore, Dihydroethidium (DHE) staining indicated a substantial increase in ROS production in AP tissues, ≈6.75‐fold higher than in normal tissues (Figure 1C,D). Additionally, immunofluorescence co‐staining of CD68 (a macrophage marker^[^ ^18^ ^]^) and STING proteins demonstrated higher expression of both markers in AP tissues, with STING primarily localized in macrophages (Figure 1E,F). These findings confirm the presence of a ROS burst during AP progression, accompanied by mt‐DNA leakage from PACs and elevated STING expression in macrophages.

To address these pathological changes, we synthesized mTWNDs as efficient mtROS scavengers by reducing phosphotungstic acid using TA and PDA under alkaline conditions. Transmission electron microscopy (TEM) examination indicated that the hydrodynamic diameter of mTWND is 10.9 nm and is uniformly distributed in an aqueous solution (Figure 1G). As anticipated, mTWNDs exhibited a negatively charged surface (−32.1 mV), attributed to TA and PDA modification (Figure 1S). To evaluate the ROS scavenging efficiency of mTWNDs, we used the predeveloped ultrasmall tungsten‐based nanodrugs (TWNDs), composed solely of polyoxometalate and TA, as a control.^[^ ^8^ ^]^ X‐ray photoelectron spectroscopy (XPS) examination indicated that mTWNDs consisted of four elements: oxygen (O), nitrogen (N), carbon (C), and tungsten (W). In contrast, TWNDs without PDA modification contained only O, C, and W (Figure 1H; Figure S2, Supporting Information). Additionally, the fine N1s peak in the XPS spectrum indicated that the N element in mTWNDs was primarily pyrrolidone N (binding energy: 400.3 eV), confirming the successful modification of PDA on mTWNDs (Figure 1I). Moreover, the R_2_NH structure in mTWNDs exhibited unique hydrogen bonding properties and functioned as a key active group for modern drug formulations. This R_2_NH functional group demonstrated high drug‐loading efficiency and controlled release capability, preventing burst release and ensuring sustained plasma drug concentration.^[^ ^19^ ^]^ These features align perfectly with the therapeutic requirement for prolonged efficacy in AP treatment. Fourier transform infrared spectroscopy (FTIR) results verified successful TA modification of mTWNDs (Figure 1J; Figure S3, Supporting Information). The UV–Vis–NIR spectra showed characteristic absorption peaks ranging from visible to NIR region (Figure S4, Supporting Information), indicating a high proportion of W^5+^ in mTWNDs because of charge transfer. XPS fine peaks of W4f revealed that the W elements in mTWNDs primarily consisted of W^5+^ and W^6+^, with W^5+^ accounting for 79.9% of the total tungsten content (Figure 1K). In comparison, TWNDs exhibited a W^5+^ content of only 53.9%, suggesting that PDA modification significantly increased the proportion of W^5+^ in mTWNDs (Figure 1L; Figures S5 and S6, Supporting Information). Since the ROS scavenging capability of mTWNDs is determined by their W^5+^ content, this enhancement is critical for their therapeutic performance. Finally, XPS assays were administered to evaluate the reaction of mTWNDs with various ROS, including hydrogen peroxide (H_2_O_2_), superoxide anion radicals (O_2_^•−^), hydroxyl radicals (·OH), and peroxynitrite (ONOO^−^). Upon reaction with H_2_O_2_, W^5+^ in mTWNDs was oxidized to W^6+^ (Figure 1M), while the spectral peaks of carbon (C), nitrogen (N), and oxygen (O) remained unchanged (Figure 1N; Figure S7, Supporting Information). Similar results were observed after reactions of mTWNDs with ·OH (Figure S8, Supporting Information), O_2_^•−^ (Figure S9, Supporting Information), and ONOO^−^ (Figure S10, Supporting Information), confirming the elimination of various ROS by mTWNDs via W^6+^/W^5+^ redox cycling. As anticipated, the nitro blue tetrazolium (NBT) assay demonstrated that mTWNDs effectively scavenged O_2_^•−^ with a superoxide dismutase (SOD) activity of ≈324 U mg^−1^, which was 1.60 times higher than that of TWNDs (203 U mg^−1^) (Figure 1O; Figure S11, Supporting Information). Similarly, mTWNDs exhibited superior efficacy in eliminating ·OH (Figure S12, Supporting Information), ONOO^−^ (Figure S13, Supporting Information), and H_2_O_2_ (Figure S14, Supporting Information) compared to TWNDs. To evaluate the stability of mTWNDs under oxidative stress conditions, we analyzed their appearance and UV spectral absorption. The results demonstrated the stability and excellent ROS scavenging ability of mTWNDs. The results of UV spectral absorption showed that their characteristic spectral absorption peaks‐attributable to charge transfer from W^5+^ to W^6+^ utilized tungsten‐oxygen bond bridging‐showed significant attenuation (Figure S15, Supporting Information). This phenomenon further confirms that mTWNDs oxidize W^5+^ to W^6+^ to scavenge ROS. These findings validate the successful synthesis of mTWNDs and highlight that the incorporation of melanin significantly enhances their ROS scavenging efficiency.

Next, we investigated the binding ability of mTWNDs to type III collagen and TOM20, using glycogen and albumin as controls. The mTWNDs were labeled with fluorescein isothiocyanate (FITC) (Figure S16, Supporting Information), and plates were coated with albumin, glycogen, collagen III, and TOM20 before immersion in the mTWNDs‐FITC solution. As shown in Figure 1P, mTWNDs exhibited strong binding to collagen III and TOM20‐coated plates, with fluorescence intensities ≈20 times higher than those observed for glycogen‐ and albumin‐coated plates (Figure S17, Supporting Information). Furthermore, binding energy calculations using Pyrx Vina revealed that the binding energies of TA to albumin and glycogen were relatively weak (−0.8 kcal mol^−1^), whereas the binding energies to collagen III and TOM20 were significantly higher at −8.5 and −8.9 kcal mol^−1^ (Figure 1Q; Figure S18, Supporting Information). These results collectively confirm the successful synthesis of mTWNDs. The incorporation of PDA greatly enhanced the ROS scavenging efficiency of mTWNDs, while TA modification conferred the ability to bind specifically to TOM20 and type III collagen, thereby optimizing their therapeutic potential.

### 2.2 mTWNDs Target Damaged Pancreatic Tissue for AP Treatment

**Figure 2:**
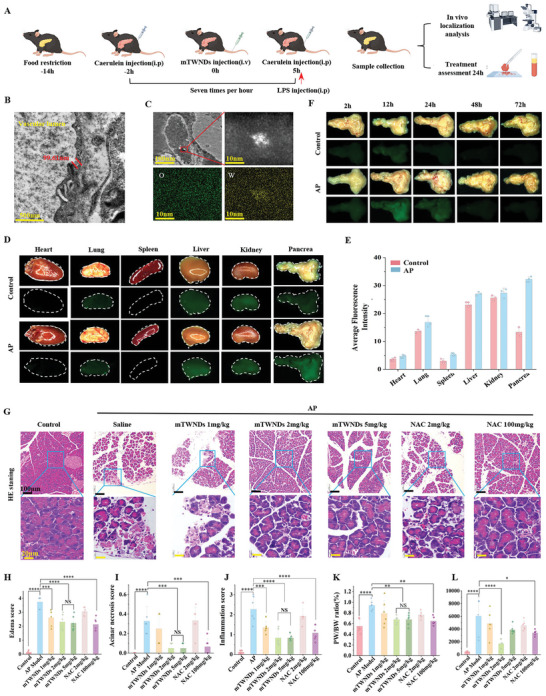
mTWNDs Target Damaged Pancreatic Tissue for AP Treatment. A) Diagrammatic representation of the development of the AP model and drug treatment protocol. B) TEM images of pancreatic capillaries in AP mice. Scale bar: 500 nm. C) HAADF‐STEM images of pancreatic capillaries in AP mice. D) Stereofluorescence images showing the distribution of mTWNDs‐FITC in various organs 12 h post‐injection. E) Quantitative analysis of stereofluorescence data for mTWNDs‐FITC distribution in different organs in vivo 12 h post‐injection (*n =* 4 animals per group). F) Time‐course analysis of mTWNDs‐FITC distribution in the pancreas at different time points. G. HE staining of pancreatic tissues from each treatment group. H–J) Quantitative scoring of edema (H), acinar necrosis (I), and inflammatory infiltration (J) based on HE staining (*n =* 6 animals per group). K,L) Pancreas‐to‐body weight ratio (PW/BW) (K) and serum AMY levels (L) in normal and AP mice at 24 h following various treatments (*n =* 6 animals in every group). Data expressed as mean ± S.D. One‐way ANOVA was utilized for statistical analysis. *P*‐values were expressed as follows: ^*^ for *p <* 0.05, ^**^ for *p <* 0.01, ^***^ for *p <* 0.001, ^****^ for *p <* 0.0001 versus AP group, intraperitoneal injection as i.p., intravenous injection as i.v.

The BPB holds a pivotal position in regulating drug delivery from the vasculature to pancreatic lesions.^[^ ^20^ ^]^ In AP, the impaired BPB facilitates mTWNDs access to damaged pancreatic tissue due to glycosylation loss and exposure to type III collagen. To investigate this, we established a mouse AP model using intraperitoneal caerulein injection (**Figure** **2**A). All mice were deprived of food for 12 h and subsequently administered seven intraperitoneal administrations of caerulein (100 µg kg^−1^ per hour), followed by a final simultaneous administration of lipopolysaccharide (10 mg kg^−1^). To evaluate the optimal timing for pharmacological intervention with mTWNDs, we administered the drug intravenously at different time points and assessed outcomes using serum amylase levels and HE scores. The results indicated that the administration of mTWNDs after the second caerulein injection was the most effective intervention time (Figures S19–S21, Supporting Information). Following the second injection of caerulein, mice received either mTWNDs or saline via tail vein injection, and pancreatic tissues were collected for TEM observation 24 h later. In normal mice, pancreatic capillaries appeared morphologically intact, with tightly connected endothelial cells. In contrast, the AP group exhibited significant endothelial cells and vascular basement membrane swelling. Endothelial cells in this group lost their tight junctions, forming gaps of varying sizes, with an average width of ≈99.61 nm (Figure 2B; Figure S22, Supporting Information). Given the particle size of mTWNDs (10.9 nm), which is substantially smaller than the endothelial gaps observed after injury, this size allows for the potential enrichment of mTWNDs in AP tissues. As anticipated, a large distribution of particles was observed both inside and outside the capillaries of pancreatic tissues in the AP group. High‐angle annular dark field (HAADF) scanning transmission electron microscopy (STEM) further verified these particles as mTWNDs (Figure 2C). To investigate the biodistribution of mTWNDs, healthy mice and AP mice were injected with mTWNDs‐FITC. Mice were euthanized using CO_2_ at 2, 12, 24, 48, and 72 h following administration, and tissues from critical organs were collected for stereofluorescence microscopy. In healthy mice, mTWNDs‐FITC primarily accumulated in the kidneys and liver, with minimal distribution in the pancreas, heart, lungs, and spleen. Notably, in AP mice, fluorescent nanomedicines predominantly accumulated in the pancreas, followed by the kidneys, liver, lungs, spleen, and heart (Figure 2D,E). These findings confirm the ability of mTWNDs to effectively target the damaged pancreas in AP mice. Interestingly, mTWNDs exhibited relatively high accumulation in the kidneys and liver in both healthy and AP mice, indicating elimination via the urinary system and the hepatic‐fecal pathway. Furthermore, the concentration of mTWNDs‐FITC in the pancreas peaked 12 h post‐injection, followed by a gradual decline over time (Figure 2F; Figure S23, Supporting Information). Similarly, the concentration of mTWNDs‐FITC in other organs began to decline gradually after 12 h and nearly disappeared by 72 h (Figures S24–S26, Supporting Information), indicating that mTWNDs exhibit good biodegradability and can be metabolized effectively in vivo. This biodegradability may be attributed to the chemical similarity between tungsten (W) and molybdenum (Mo), both of which belong to group 6 of the chemical elements table. Molybdenum is an essential trace element in mammals and serves as a central component of numerous key enzymes that provide their active centers.^[^ ^21^ ^]^ Based on this similarity, tungsten may also be efficiently excreted via metabolic pathways utilized by molybdenum in living organisms.

Considering the strong ROS scavenging ability of mTWNDs and their targeted effect on the injured pancreas, we evaluated their therapeutic efficacy in AP mice. NAC, a clinically approved and experimentally validated antioxidant drug effective against AP,^[^ ^22^ ^]^ was used as a control in this study. Hematoxylin‐eosin (HE) staining was performed to assess pancreatic tissue damage across different treatment groups. In AP mice, PACs exhibited pronounced edema and necrosis, accompanied by significant inflammatory cell infiltration. The therapeutic efficacy of mTWNDs was dose‐dependent. At mTWNDs concentrations of 2 mg kg^−1^ and 5 mg k^−1^g, the damage to pancreatic tissue was significantly alleviated, whereas a dose of 1 mg kg^−1^ showed no therapeutic effect (Figure 2G). HE scoring further confirmed that mTWNDs reduced pancreatic tissue edema (Figure 2H), necrosis (Figure 2I), and inflammatory infiltration (Figure 2J) in AP mice. Additionally, the pancreas‐to‐body weight (PW/BW) ratio and serum amylase (AMY) levels demonstrated that mTWNDs at 2 mg kg^−1^ were effective in ameliorating AP symptoms, achieving efficacy comparable to NAC at the concentration of 100 mg kg^−1^‐≈1/50th a concentration required for NAC to be effective. Notably, NAC at this low dose had no therapeutic effect (Figure 2K,L and Figure S27, Supporting Information). Based on the principle of administering the minimum effective dose, we selected 2 mg kg^−1^ of mTWNDs for subsequent studies.

### 2.3 mTWNDs Target mtROS Scavenging to Inhibit Apoptosis

**Figure 3:**
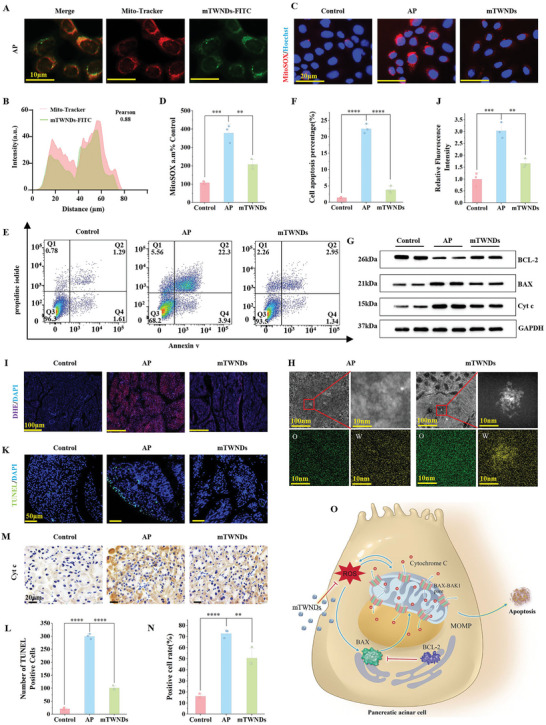
mTWNDs Target Scavenging of mtROS to Inhibit Apoptosis. A) Results of mTWNDs‐FITC intracellular localization experiments. Scale bar: 10 µm. B) Pearson coefficient analysis of (A). C,D) Immunofluorescence staining and quantification of mitochondrial superoxide in PPACs. Scale bar: 20 µm. E) Flow cytometry detection of apoptosis of PPACs across different groups. F) Quantitative analysis of flow cytometry in (E). G) WB images of BCL‐2, BAX, and Cyt c proteins in each group of PPACs. H) HAADF‐STEM images of mTWNDs in PACs mitochondria. I) DHE staining results for each group of samples. Scale bar: 100 µm. J) Quantitative analysis of DHE staining in (I). K) TUNEL, DAPI staining, and merged images for each group of samples. Scale bar: 50 µm. L) Quantitative analysis of TUNEL staining in (K). M) Immunohistochemical results of Cyt c in pancreatic tissues across different groups. Scale bar: 20 µm. N) Quantitative statistical analysis of Cyt c immunohistochemistry in (M). O) Schematic representation of mTWNDs scavenging ROS to reduce mitochondria‐dependent apoptosis. Data were displayed as mean ± S.D., obtained from three separate experiments (*n =* 3). One‐way ANOVA was utilized for statistical analysis. *P*‐values were expressed as follows: ^*^ for *p <* 0.05, ^**^ for *p <* 0.01, ^***^ for *p <* 0.001, ^****^ for *p <* 0.0001 versus AP group.

Primary pancreatic acinar cells (PPACs) were isolated from the pancreatic tissues of healthy mice to investigate the mitochondria‐targeting ability of mTWNDs. Mitochondria were labeled with Mito‐tracker, and cells were incubated with mTWNDs‐FITC to track their distribution within PPACs. As shown in **Figure** **3**A,B, mTWNDs strongly targeted the mitochondria of PPACs, as evidenced by the strong Pearson's correlation coefficient of 0.88. This mitochondria‐targeting capability was attributed to the strong affinity of TA on the mTWNDs surface for TOM20. To further evaluate the ability of mTWNDs to scavenge mtROS, cholecystokinin (CCK)‐induced PPACs were assessed using the MitoSOX red probe assay. Fluorescence intensity of MitoSOX was significantly increased in CCK‐induced PPACs‐≈3–4 times higher than in normal controls‐indicating elevated mtROS levels. Notably, mTWNDs treatment markedly reduced MitoSOX fluorescence intensity in CCK‐induced PPACs (Figure 3C,D), suggesting efficient mitochondria‐targeted ROS scavenging by mTWNDs. Additionally, flow cytometry analysis confirmed the efficacy of mTWNDs in eliminating mtROS in CCK‐induced PPACs (Figure S28, Supporting Information). Next, the Annexin V‐FITC/PI flow cytometry analysis was utilized to perform on PPACs under different treatment conditions to confirm that mTWNDs effectively inhibited excessive apoptosis in PPACs (Figure 3E,F). Western blot (WB) analysis further demonstrated that mTWNDs significantly increased the development of B‐cell lymphoma‐2 (BCL‐2), while decreasing the expression of BCL‐2‐associated X (BAX) as well as the release of Cyt c in PPACs (Figure 3G; Figure S29, Supporting Information).

HAADF‐STEM analysis of pancreatic tissues from each group confirmed the widespread distribution of mTWNDs on the mitochondria of PACs (Figure 3H). The amount of ROS in pancreatic tissues were further assessed using DHE staining. As expected, the sample tissues of the AP group exhibited significant ROS generation, whereas mTWNDs demonstrated a robust ROS scavenging effect (Figure 3I,J). The anti‐apoptotic effects of mTWNDs were further validated through Terminal Deoxynucleotidyl Transferase‐Mediated dUTP Nick End Labeling (TUNEL). Lesion tissues of AP exhibited a high apoptosis rate of 41.25%, which was significantly reduced to 13.63% following mTWNDs treatment (Figure 3K,L). Immunohistochemical analysis revealed that Cyt c expression intensity in the AP group was ≈4.22‐fold stronger than in the normal control group. Administration of mTWNDs significantly reduced Cyt c expression in AP pancreatic tissues (Figure 3M,N). Additionally, WB analysis of pancreatic tissue homogenates confirmed that mTWNDs promoted BCL‐2 expression while reducing BAX expression and Cyt c release, thereby inhibiting apoptosis (Figures S30 and S31, Supporting Information). Collectively, these results demonstrate that mTWNDs effectively inhibit apoptosis in AP by targeting mitochondria and disrupting the vicious cycle of mtROS‐mitochondrial damage‐Cyt c release (Figure 3O).

### 2.4 mTWNDs Inhibit Macrophage Activation via cGAS/STING

**Figure 4:**
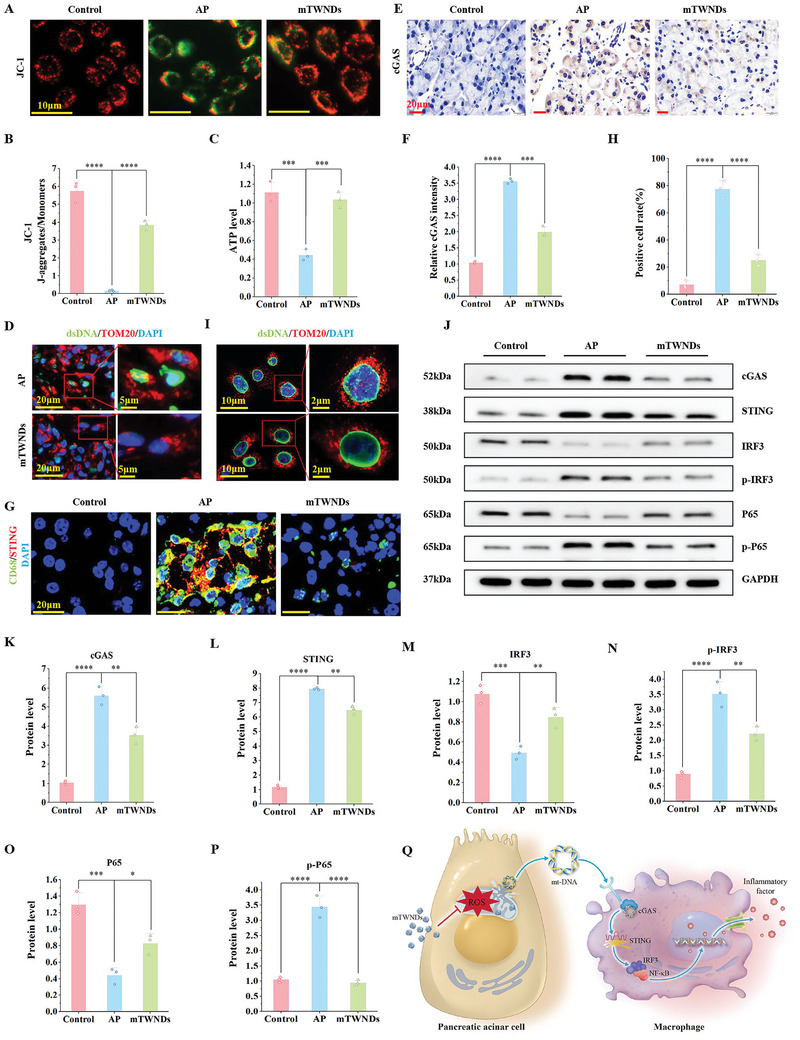
mTWNDs Block the cGAS/STING Pathway by Protecting Mitochondria. A) Immunofluorescence staining of JC‐1 in PPACs among various groups. Scale bar: 10 µm. B) Statistical analysis of immunofluorescence for JC‐1 from (A). C) ATP production rate in PPACs across different groups. D) Immunofluorescence staining of dsDNA, TOM20, and DAPI in pancreatic tissues of mice across different groups. E) Immunohistochemistry of cGAS in pancreatic tissues among various groups. Scale bar: 20 µm. F) Statistical analysis of cGAS immunohistochemistry from (E). G) Immunofluorescence staining of CD68 and STING in pancreatic tissues across different groups. Scale bar: 20 µm. H) Statistical analysis of CD68 and STING immunofluorescence from (G). I) Immunofluorescence staining of dsDNA and TOM20 in PPACs across different groups. J) WB results for cGAS/STING pathway‐related proteins in macrophages across different groups. K–P) Quantitative analysis of WB results for cGAS (K), STING (L), IRF3 (M), p‐IRF3 (N), P65 (O), and p‐P65 (P) proteins. Q. Schematic representation of mTWNDs inhibiting the cGAS/STING pathway by protecting mitochondria. Data were displayed as mean ± S.D., obtained from three separate experiments (*n =* 3). One‐way ANOVA was utilized for statistical analysis. *P*‐values were expressed as follows: ^*^ for *p <* 0.05, ^**^ for *p <* 0.01, ^***^ for *p <* 0.001, ^****^ for *p <* 0.0001 versus AP group.

TEM revealed normal mitochondrial morphology with clearly defined cristae in healthy control mice. In contrast, mitochondria in AP mice appeared swollen and ruptured, with fragmented cristae containing the mitochondrial respiratory chain complex and a sparse mitochondrial matrix. As previously described, mTWNDs can effectively enter PACs via the BPB in AP mice, targeting mitochondria. Following mTWND treatment, the mitochondrial morphology of PACs in AP mice was restored to a near‐normal state (Figure S32, Supporting Information). In AP, excessive mtROS damage disrupts the mitochondrial membrane, leading to a reduction in mitochondrial membrane potential (MMP). It was observed that MMP in PPACs was significantly reduced following CCK‐induced treatment by employing the JC‐1 probe. Treatment with mTWNDs significantly restored MMP to near‐normal levels (**Figure** **4**A,B; Figure S33, Supporting Information). Mitochondrial function, assessed through ATP production, was also significantly impaired in CCK‐induced PPACs. The mTWND treatment restored ATP production in PPACs to near‐normal levels (Figure 4C). Immunofluorescence analysis of pancreatic tissues revealed substantial mt‐DNA leakage in AP mice, while mt‐DNA leakage was nearly absent in mTWNDs‐treated mice (Figure 4D). These findings were consistent with observations in human pancreatic tissue. Immunohistochemical analysis further demonstrated significantly elevated levels of cGAS and STING in AP mice compared to healthy controls, indicating initiation of the cGAS/STING signaling cascade in pancreatic tissues. Notably, mTWNDs treatment markedly inhibited cGAS and STING activation in pancreatic tissues (Figure 4E,F; Figure S34, Supporting Information). WB analysis of pancreatic tissue homogenates corroborated these findings. The expression levels of cGAS/STING cascade activation‐associated molecules were markedly elevated in AP mice. Treatment with mTWNDs reduced cGAS and STING expression and inhibited the phosphorylation of interferon regulatory factor 3 (IRF3) (p‐IRF3) and P65 (p‐P65), further demonstrating suppression of the cGAS/STING signaling cascade (Figures S35 and S36, Supporting Information). Subsequently, immunofluorescence co‐staining for CD68 and STING was performed on mouse pancreatic tissues from different groups. Consistent with findings from human tissues, STING was predominantly expressed in macrophages rather than PACs (Figure 4G,H), suggesting that DNAs released by PACs during AP may activate the STING pathway in macrophages.

At the cellular level, it was further verified that mt‐DNA released by PACs primarily activates STING signaling in macrophages (RAW264.7). PPACs were extracted from mouse pancreatic tissues, with the AP group treated using CCK for induction, and mTWNDs added to the drug group for co‐culture. After 18 h, supernatants from each group of PACs were collected and used to culture macrophages to study STING activation. First, the distribution of mt‐DNA in PPACs was observed via co‐staining with dsDNA, TOM20, and DAPI. As shown in Figure 4I, significant mt‐DNA release was observed in the mitochondria of CCK‐induced PPACs, whereas treatment with mTWNDs markedly inhibited this process. Next, macrophages from different treatment groups were collected for WB analysis to examine the levels of cGAS/STING signaling‐associated proteins. The supernatant from the AP group induced the expression of cGAS and STING in macrophages as well as enhance the amount of p‐IRF3 and p‐P65, indicative of pathway activation. In contrast, macrophages cultured with supernatants from the mTWNDs‐treated group exhibited suppressed expression of cGAS/STING‐related proteins, reaching levels comparable to those in the normal control group (Figure 4J–P). Overall, the above findings confirm that mTWNDs protect PACs mitochondria, reduce mt‐DNA leakage, along block activation of the STING‐associated inflammation in macrophages (Figure 4Q).

### 2.5 mTWNDs Reverse Macrophage Inflammation

**Figure 5:**
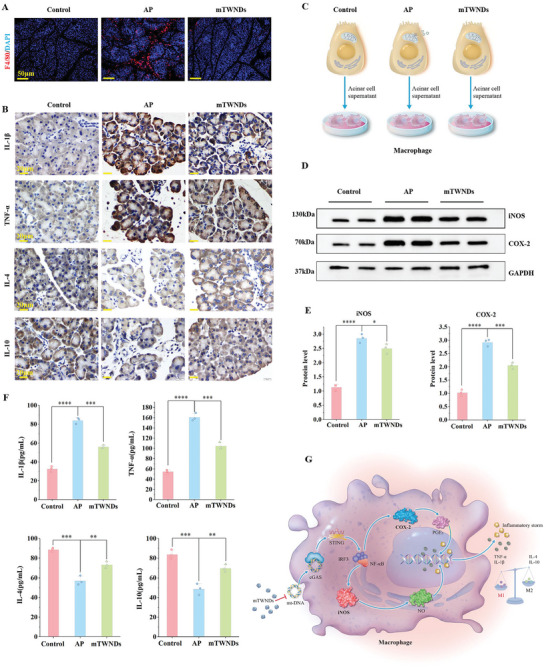
mTWNDs Reverse Macrophage Inflammation. A) F4/80 staining of pancreatic tissue from various groups. Scale bar: 50 µm. B) Immunohistochemical labeling images of TNF‐α, IL‐1β, IL‐4, and IL‐10 in pancreatic tissues across various groups. Scale bar: 20 µm. C) Schematic representation of macrophage induction by supernatants from PPACs in different treatment groups. D,E) WB analysis (D) and quantitative evaluation (E) of COX‐2 and iNOS protein levels in RAW264.7 cells across various groups. F) ELISA assay of TNF‐α, IL‐1β, IL‐4, and IL‐10 in RAW264.7 across different groups. G) Schematic representation of inflammation inhibition in macrophages by mTWNDs. Data were displayed as mean ± S.D., in vivo: three animals in every group, in vitro: three separate experiments (*n =* 3). One‐way ANOVA was utilized for statistical analysis. *P*‐values were expressed as follows: ^*^ for *p <* 0.05, ^**^ for *p <* 0.01, ^***^ for *p <* 0.001, ^****^ for *p <* 0.0001 versus AP group.

It is widely confirmed that the cGAS/STING cascade triggers the NF‐κB signaling pathway, which stimulates the secretion of inflammatory elements. As demonstrated above, mt‐DNA released from PACs activates the cGAS/STING cascade in macrophages during AP, resulting in increased P65 expression. To further evaluate the impact of mTWNDs on macrophage‐mediated inflammation, we examined macrophage infiltration and polarization in pancreatic tissues. F4/80 immunostaining of pancreatic tissues revealed significant macrophage infiltration within AP mice relative to normal mice. Encouragingly, administration of mTWNDs effectively blocked macrophage accumulation in pancreatic tissue (**Figure** **5**A; Figure S37, Supporting Information). Macrophage polarization was further analyzed using tissue immunofluorescence. The results indicated that pro‐inflammatory M1‐type macrophages predominated in pancreatic tissues of the AP group. In contrast, anti‐inflammatory M2‐type macrophages were predominant after treatment with mTWNDs. These findings confirm that mTWNDs repress M1 macrophage polarization and simultaneously promote M2 macrophage polarization, thereby reducing inflammation (Figure S38, Supporting Information). Next, the levels of various inflammatory factors in tissue homogenates of each sample from each group were determined utilizing enzyme‐linked immunosorbent assay (ELISA). Factors that promote inflammation, comprising tumor necrosis factor‐α (TNF‐α) and interleukin‐1β (IL‐1β), were significantly elevated in AP mice‐3.15‐fold and 2.36‐fold higher, respectively, compared to the healthy group. In contrast, the anti‐inflammatory elements interleukin‐4 and −10 (IL‐4, IL‐10) were markedly reduced in AP mice, indicating a caerulein‐induced inflammatory storm in pancreatic tissue. As anticipated, mTWNDs treatment significantly reduced the production of pro‐inflammatory mediators (IL‐1β, TNF‐α) while increasing the amount of anti‐inflammatory mediators (IL‐4, IL‐10), demonstrating an anti‐inflammatory effect (Figure S39, Supporting Information). Immunohistochemical analysis of pancreatic tissues further confirmed that mTWNDs inhibited the expression of IL‐1β and TNF‐α while enhancing the production of IL‐4 and IL‐10, thereby mitigating inflammation (Figure 5B; Figure S40, Supporting Information).

To further explore the impact of mTWNDs on macrophage inflammation, macrophages were cultured with supernatants of PPACs from different treatment groups and subsequently subjected to immunofluorescence and WB analysis (Figure 5C). Immunofluorescence results revealed that the level of M1 polarization‐associated markers, cyclooxygenase‐2 (COX‐2), and inducible nitric oxide synthase (iNOS), was remarkably elevated in macrophages cultured with supernatants from the AP group. In contrast, therapy with mTWNDs significantly suppressed the production of COX‐2 and iNOS (Figure S41, Supporting Information). Similarly, WB analysis demonstrated increased levels of COX‐2 and iNOS in macrophages treated with medium obtained from the AP group, whereas macrophages cultured with supernatants from the mTWNDs‐treated group showed reduced expression of these markers (Figure 5D,E). ELISA results corroborated these findings, showing that the production of pro‐inflammatory factors IL‐1β and TNF‐α was elevated, while the production of anti‐inflammatory factors IL‐10 and IL‐4 was significantly reduced in macrophages cultured with supernatants from the AP group. Treatment with mTWNDs effectively reversed these inflammatory markers, leading to marked improvements in inflammation (Figure 5F). In conclusion, mTWNDs reduced macrophage infiltration, inhibited M1 macrophage polarization, and attenuated inflammation, achieving therapeutic effects in AP (Figure 5G).

### 2.6 Biocompatibility of mTWNDs

**Figure 6:**
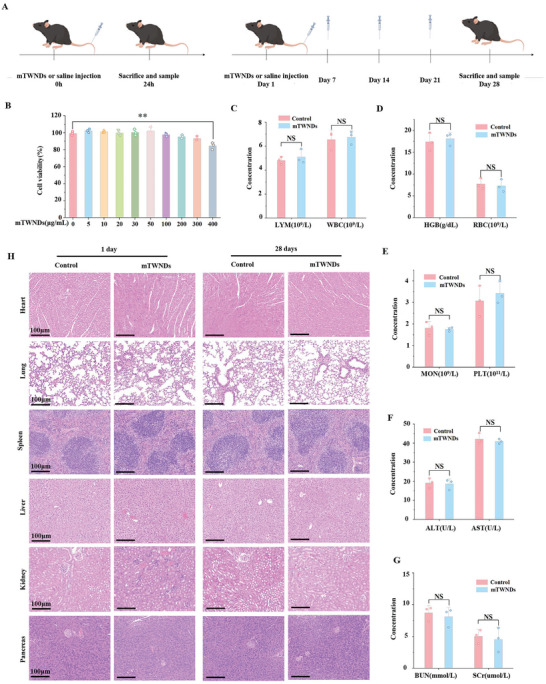
Safety and Biocompatibility Analysis of mTWNDs. A) Diagrammatic depiction of the experimental program for biocompatibility analysis of mTWNDs. B. In vitro cytotoxicity analysis of mTWNDs on PPACs. C–E) Serum levels of LYM and WBC (C), HGB and RBC (D), and monocytes (MON) and PLT (E) in normal mice 24 h following intravenous injection of saline or mTWNDs. F,G) Serum levels of hepatic function indices (F) and renal function indices (G) in normal mice 24 h following intravenous injection of saline or mTWNDs. H. HE staining on vital organs (heart, pancreas, liver, spleen, lungs, and kidneys) 1‐ and 28 days following injection of mTWNDs. Scale bar: 100 µm. Data were displayed as mean ± S.D., obtained from three separate experiments (*n =* 3). The method of statistical analysis was the unpaired t‐tests. *P*‐values were expressed as follows: ^*^ for *p <* 0.05, ^**^ for *p <* 0.01, ^***^ for *p <* 0.001, ^****^ for *p <* 0.0001 versus the control group, no significance as NS.

In the end, the biocompatibility of mTWNDs was assessed to evaluate their potential for clinical translation (**Figure** **6**A). At the cellular level, mTWNDs exhibited negligible toxicity toward PPACs, with only slight changes in cell viability observed at concentrations as high as 400 µg mL^−1^‐far exceeding the effective application concentration (Figure 6B). These results indicate that mTWNDs possess excellent safety in terms of cells. At the animal level, the safety of mTWNDs was further validated. Whole blood samples were collected 24 h after drug injection to evaluate complete blood counts, liver and kidney functions. Results demonstrated that mTWNDs had no significant effects on red blood cells (RBCs), white blood cells (WBCs), hemoglobin (HGB), platelets (PLTs), and lymphocytes (LYM) (Figure 6C–E). Furthermore, high‐dose mTWNDs treatment (20 mg kg^−1^) did not impact liver function, as indicated by stable alanine aminotransferase (ALT) and aspartate aminotransferase (AST) indexes (Figure 6F). Kidney function remained unaffected, as evidenced by normal blood urea nitrogen (BUN) and serum creatinine (Scr) indexes (Figure 6G). To assess the toxicity of mTWNDs, normal mice received a high dose of mTWNDs (20 mg kg^−1^, 10 times the effective treatment dose) or saline. Histological examinations of the heart, pancreas, lungs, liver, spleen, and kidneys were conducted 1 and 28 days after injection, using HE, Masson, and Congo red staining. The morphology and structure of vital organs remained intact, with no observed histological abnormalities such as cellular wrinkling, amyloidosis, or fibrosis (Figure 6H; Figures S42 and S43, Supporting Information). These findings confirm that mTWNDs exhibit outstanding biocompatibility, underscoring the promise of clinical applications.

## 3 Discussion

The BPB is a dynamic, highly selective filtration interface that restricts the penetration of most small molecules and all large molecules, significantly influencing drug distribution and therapeutic efficacy in the pancreas.^[^ ^6^, ^23^ ^]^ Overcoming this barrier remains a critical challenge in developing effective treatments for pancreatic diseases. A few strategies have been proposed to address this issue, including the design of novel osmotic agents that enhance drug permeability across the BPB.^[^ ^24^ ^]^ However, nonspecific disruption of the BPB's physiological barrier function can contribute to unintended damage to pancreatic tissues. Invasive delivery methods, such as regional arterial perfusion, have also been developed to bypass the BPB. While these methods can facilitate direct drug delivery to pancreatic tissues, they pose risks such as bleeding, infection, and significant discomfort for patients.^[^ ^25^ ^]^ More importantly, these methods of facilitating drug delivery to the pancreas are nonspecific and fail to effectively target the lesion site for therapeutic efficacy. This limitation has driven the emergence of various nanomedicines for inflammatory diseases, propelled by advancements in materials science and nanotechnology. Nanomedicines are generally capable of passively targeting damaged sites due to their small size.^[^ ^26^, ^27^ ^]^ Furthermore, rational surface modifications of nanomedicines enhance their surface charge, biocompatibility, and stability in biological fluids, prolonging their circulation time in the bloodstream.^[^ ^17^, ^28^ ^]^ However, currently developed nanomedicines for the treatment of AP remain suboptimal due to their incompatibility with the damaged BPB and challenges in achieving mitochondrial targeting.^[^ ^29^, ^30^ ^]^ The unique properties of mTWNDs, developed using TA, address these challenges. The high binding affinity of TA to collagen III, combined with the extremely small particle size of mTWNDs, allows these nanomedicines to effectively recognize and traverse damaged BPBs. This capability forms the foundation for mTWNDs’ precise and efficient treatment of AP.

The mouse AP model demonstrated a ROS storm in pancreatic tissue, with excessive ROS‐inducing apoptosis closely linked to Cyt c release. Cyt c, a peripheral protein on the inner mitochondrial membrane, holds a central position in mitochondrial oxidative phosphorylation by the transfer of electrons, thus supporting cellular energy production.^[^ ^31^ ^]^ Excessive ROS generation increases the production of the pro‐apoptotic agent BAX, which forms oligomers with BCL‐2‐antagonist/killer 1 (BAK1), inhibiting the anti‐apoptotic agent BCL‐2. This oligomerization of BAX and BAK1 drives mitochondrial outer membrane permeabilization (MOMP), causing Cyt c release out of mitochondria and subsequent apoptosis.^[^ ^32^ ^]^ Moreover, the released Cyt c amplifies ROS production in the respiratory chain, perpetuating a vicious cycle of mtROS‐BAX‐mitochondrial damage‐Cyt c release‐apoptosis.^[^ ^32^, ^33^ ^]^ Notably, PDA significantly enhanced the antioxidant capacity of mTWNDs. Additionally, the strong binding affinity of TA to TOM20 conferred mTWNDs with robust mitochondrial targeting capabilities. These properties enabled mTWNDs to effectively scavenge ROS within the mitochondria of PACs. Consequently, mTWNDs specifically eliminated mtROS, reduced Cyt c release, and inhibited apoptosis in PACs.

Impaired mitochondrial function has been established as a central contributor to the pathological process of AP.^[^ ^34^ ^]^ In addition to being a primary generator of endogenous ROS, mitochondria produce various damage‐associated molecular patterns (DAMPs), particularly mt‐DNA.^[^ ^35^, ^36^ ^]^ DNA released into circulation from damaged PACs serves as a biomarker for AP. During apoptosis, mt‐DNA leaks out of mitochondria into the cytoplasm as a form of new cytoplasmic DNA via BAK1/BAX oligomers on the outer mitochondrial membrane, alongside the outflow of Cyt c.^[^ ^37^ ^]^ The presence of free DNA in the circulation is considered a critical indicator of cellular damage and inflammation. Among DNA sensors, cGAS specifically recognizes various DNA types, with a particular affinity for mt‐DNA. The binding of cGAS to mt‐DNA activates the STING cascade, which promotes IRF3‐mediated synthesis of type I interferons (IFNs) and NF‐κB‐mediated synthesis of pro‐inflammatory factors. This activation drives an inflammatory cascade, amplifying the immune response.^[^ ^38^, ^39^ ^]^ Macrophages, as innate immune cells with dynamic phenotypic plasticity, hold an important position in the progression of AP.^[^ ^40^ ^]^ STING proteins directly influence the generation of IFNs, promoting M1 polarization of macrophages and affecting the recruitment of undifferentiated macrophages.^[^ ^41^, ^42^, ^43^ ^]^ The present study confirms that mt‐DNA released from PACs during AP activates macrophages via the STING pathway, promoting M1 polarization and contributing to an inflammatory storm. More importantly, mTWNDs reduced the outflow of mt‐DNA, effectively blocking the initiation of the cGAS/STING pathway in macrophages. This intervention inhibited macrophage aggregation and M1 polarization, significantly alleviating AP‐associated inflammation.

According to the findings of this study, TA‐based modifications not only enable mTWNDs to traverse the damaged BPB more efficiently but also endow these nanomedicines with mitochondrial targeting capabilities. The highly effective ROS‐scavenging properties of mTWNDs provide a promising strategy for treating AP. However, this study has several limitations, particularly concerning in vivo animal models and in vitro cellular experiments. For the animal model, we employed the widely accepted cerulein‐induced pancreatitis model in mice. While this animal model can simulate a disease environment similar to that of the human body, challenges remain regarding the stability and consistency of model replication. These challenges may impact the evaluation and reproducibility of drug efficacy. Additionally, differences in drug responses between species pose limitations to directly extrapolating findings from animal models to clinical applications. Furthermore, only male mice were used in this study to ensure experimental consistency and maneuverability. However, gender‐specific differences in pharmacodynamics and pharmacokinetics may influence drug efficacy and toxicity, highlighting the need for future studies to explore these variables. In the in vitro cell model, although PPACs were extracted, the culture conditions did not fully replicate the in vivo environmental and physiological conditions. This limitation reduces the physiological relevance of the findings, as drug effects and toxicity observed in vitro may not fully align with in vivo outcomes. As a next step, we aim to deepen our understanding of the pathophysiological differences between human patients and animal models to further optimize mTWNDs for improved clinical translation.

## 4 Conclusion

In this study, mitochondria‐targeted ROS scavengers, mTWNDs, were successfully developed using TA to mediate the assembly of phosphotungstic acid and PDA into nanoscale complexes. The results demonstrated that mTWNDs could recognize and traverse the impaired BPB, specifically target the mitochondria of PACs and exhibit potent ROS scavenging capabilities. Consequently, mTWNDs effectively eliminated mtROS in PACs and significantly reduced apoptosis. Furthermore, mTWNDs mitigated the release of mt‐DNA in PACs, inhibited the activation of cGAS/STING pathway in macrophages, and reversed macrophage‐associated inflammation. At an extremely low dose (2 mg k^−1^g), mTWNDs alleviated symptoms in AP mice, achieving a therapeutic effect that was significantly superior to that of the clinically applied NAC. This study addresses the challenge of specifically targeting the damaged BPB and mitochondria for the effective treatment of AP. The findings confirm that mTWNDs are ideal candidates for targeting the scavenging of mitochondrial ROS to treat a variety of inflammatory diseases.

## 5 Experimental Section

### Materials Details

### Synthesis of mTWNDs, TWNDs, and mTWNDs‐FITC

Briefly, the preparation of mTWNDs was based on the principle of redox reaction. Solution 1 was obtained by dissolving phosphotungstic acid (0.18 g) in deionized water (ddH_2_O, 10 mL). Dissolve TA (0.2 g) in ddH_2_O (15 mL) to obtain Solution 2. Solution 3 consisted of 10 mL of a 7.5% sodium carbonate (Na_2_CO₃) solution. 0.1 g of dopamine hydrochloride was mixed with ddH_2_O (5 mL) to prepare Solution 4. These solutions were sequentially added to a reaction flask and stirred at 25 °C for 12 h. The product was then dialyzed for 2–3 days to remove unreacted impurities, followed by freeze‐drying for 3 days to obtain the mTWNDs (**Table** **1**).

**Table 1:** The list of materials.

Reagents	Source	Catalog
Phosphotungstic acid	Macklin	P815378
Tannic acid	Macklin	T818845
Dopamine hydrochloride	Macklin	D806618
Sodium carbonate anhydrous	Macklin	S818014
Terephthalic acid	Macklin	D835927
Riboflavin	Macklin	R817215
Methionine	Macklin	L812760
Nitro‐blue tetrazolium	Macklin	N814596
Fluorescein isothiocyanate	Macklin	F6120
Ferrous sulfate	Aladdin	F116338
Hydrogen peroxide	Sigma	216 763
Triphenyl tetrazolium chloride	Sigma	T8877
Ceruletide	MedChemExpress	HY‐A0190
Cholecystokinin	MedChemExpress	HY‐P2932
A TUNEL assay kit	Thermo Fisher Scientific	C10617
MitoSOX Red Mitochondrial Superoxide Indicator	Thermo Fisher Scientific	M36008
Malondialdehyde (MDA) Assay Kit	Thermo Fisher Scientific	EEA015
Ultrasensitive ECL chemiluminescence kit	Beyotime	P0018S
JC‐1 probe	Beyotime	C2003S
Enhanced BCA Protein Assay Kit	Beyotime	P0010
Protease and phosphatase inhibitors	Beyotime	P1046
Cell Counting Kit‐8	Beyotime	C0038
Hoechst33342	Beyotime	C1022
Antifade Mounting Medium with DAPI	Beyotime	P0131
Enhanced ATP Assay Kit	Beyotime	S0027
Mito‐Tracker Red CMXRos	Beyotime	C1035
Annexin V‐FITC/PI Apoptosis Detection Kit	Yeasen	40302ES50
Dihydroethidium	Yeasen	50102ES

Similarly, TWNDs were synthesized by mixing phosphotungstic acid (0.18 g) and TA (0.3 g) in ddH_2_O (30 mL) and fully dissolved. To create alkaline conditions, 10 mL of 7.5% Na_2_CO₃ was introduced into the mixture. Subsequently, the mixed solution was magnetically stirred continuously for 12 h. The resulting dark green reduced solution was dialyzed and freeze‐dried to produce TWNDs.

For the functionalization of mTWNDs, 40 mg of mTWNDs and 50 mg of NH_2_‐PEG‐NH_2_ were introduced into a Tris buffer solution (pH 8.5) with continuous stirring for 12 h. mTWNDs‐PEG was prepared by dialysis for 2–3 days followed by freeze‐drying for 3 days. To prepare FITC‐labeled mTWNDs, 2 mg of FITC was added to dimethyl sulfoxide to form a dose of l mg mL^−1^. Next, 10 mg of mTWNDs‐PEG was introduced into 8 mL of ddH_2_O, and the FITC solution was added. The reaction conditions for the mixture were: dark conditions, 25 °C, for a total of 8 h. The mixture was dialyzed for 1–2 days to remove unreacted impurities, followed by freeze‐drying for 3 days to obtain mTWNDs‐FITC.

### Characterization of mTWNDs

All TEM images were obtained using a high‐resolution TEM (TECNAI G2). The elemental composition of mTWNDs and TWNDs was analyzed through XPS measurements (VG ESCALAB MKII). XPS measurements were also performed before and after reactions with various ROS, containing hydrogen peroxide (H_2_O_2_), hydroxyl radical (·OH), superoxide anion (O_2_^•−^), and peroxynitrite (ONOO^−^). The C1s, N1s, O1s, and W4f peaks of mTWNDs and TWNDs were analyzed using XPSPEAK software (version 4.1). X‐ray diffraction analyses were conducted by a Bruker D8 Discover X‐ray diffraction system. The Fourier‐transform infrared (FTIR) analysis was conducted using a Bruker Vertex 70 spectrometer (resolution of 2 cm^−1^). A VARIAN CARY 50 spectrophotometer was utilized to record the UV‐Vis absorption spectra.

### Detection of O_2_^•−^ Scavenging Capacity in vitro

The O_2_^•−^ scavenging capacity of mTWNDs and TWNDs was evaluated using the NBT method. Briefly, mTWNDs at various dosages (0.5, 1, 2, 4, 8 µg mL^−1^) were combined with phosphate‐buffered saline (PBS, 0.1 m, pH 7.4, 1.5 mL), 390 µL of methionine, 22.5 µL of NBT, 6 µL of riboflavin, and deionized water. For comparison, TWNDs at concentrations of 1, 2, 4, and 8 µg mL^−1^ were used. The cuvettes containing the mixtures were subjected to UV light for ≈5 min. The O_2_^•−the^ scavenging ability of mTWNDs and TWNDs was subsequently analyzed by measuring the absorbance at 560 nm.

### Detection of ·OH Scavenging Capacity in vitro

Fluorescence spectrophotometry was utilized to measure the ·OH removal capacity of mTWNDs and TWNDs. Various concentrations of mTWNDs (125, 250, 500, 1000 ng mL^−1^) were mixed with PBS (0.01 m, pH 7.4), terephthalic acid (0.1 mm), H_2_O_2_ (1 mm), and ferrous sulfate (0.05 mm). Similarly, TWNDs at doses of 125, 250, 500, 1000, and 2000 ng mL^−1^ were utilized for comparison. The sample was exposed for 6 min at 25 °C and then assessed by measurement at an excitation wavelength of 320 nm.

### Detection of ONOO^−^ Scavenging Capacity in vitro

The ONOO^−^ scavenging capacity of mTWNDs was analyzed using pyrogallol red as the detection reagent. A mixture containing the solution of ONOO^−^ (1.73 mm, 9 µL), pyrogallol red (5 mm, 10 µL), and various doses of mTWNDs (0, 2, 4, 8 µg mL^−1^) was prepared and permitted to react for 15 min. Similarly, TWNDs at the same concentrations were used for comparison. The ONOO^−^ removal capacity of mTWNDs and TWNDs was assessed by scanning the ultraviolet absorption spectra.

### Detection of H_2_O_2_ Scavenging Capacity in vitro

The H_2_O_2_ scavenging capacity of mTWNDs and TWNDs was measured using UV‐Vis spectrophotometry. Different concentrations of mTWNDs (0.5, 1, 2, 4, 8 µg mL^−1^) were combined with H_2_O_2_ (1 mm) and kept in the dark conditions for 12 h. TWNDs at identical concentrations were used as controls. The scavenging ability of H_2_O_2_ was assessed by measuring UV absorption at 425 nm.

### Animal Models of Acute Pancreatitis

All male mice of the C57BL/6 strain (weighing 23 ± 2 grams, aged 6–7 weeks) employed in this research were obtained from Hunan SJA Laboratory Animal Co., Ltd. Acute pancreatitis was induced using the cerulein pancreatitis model, as described previously. Mice received up to seven hourly intraperitoneal injections of cerulein (100 µg k^−1^g).^[^ ^10^ ^]^ Control mice received an equal amount of saline. Mice were executed 12 or 24 h following the first injection of cerulein (or saline). The development of pancreatitis was confirmed by measuring serum AMY levels and analyzing histological changes in hematoxylin‐eosin (HE)‐stained pancreatic tissue sections. Mice were injected with the drug of interest 2 hours after the initial Cerulein injection for further study. All animal studies were conducted under the approval protocols of the Institutional Animal Care and Use Committee (IACUC) from Xiangya Hospital, Central South University, China.

### Treatment of AP Mice

Following the second cerulein injection, each group of mice was subjected to different treatments. Group 1 consisted of normal mice administered with saline. Group 2 comprised AP mice administered with saline. Groups 3, 4, and 5 were AP mice administered with mTWNDs at dosages of 1, 2, and 5 mg k^−1^g in 200 µL of saline, respectively. Groups 6 and 7 were AP mice treated with NAC at dosages of 2 and 100 mg k^−1^g in 200 µL of saline, respectively. Each group included six mice (*n =* 6). All reagents used for treatment or control were administered via tail vein injection.

### Evaluation of Treatment Effects on AP Status

Mice were euthanized 24 h following various treatments. The treatment effectiveness of mTWNDs in the AP model was investigated using AMY tests and HE staining. The final body weight of the mice and the weight of the pancreas were measured, and the pancreas‐to‐body weight ratio was calculated. Samples of blood were collected and centrifuged at 2000 g ≈15 min to extract the serum. AMY were analyzed using an automatic biochemistry analyzer (Chemray 240, Radu Life Sciences). Subsequently, pancreatic samples were fixed with paraformaldehyde, paraffin‐embedded, sectioned, and finally stained with HE. The remaining tissue was stored at −80 °C for further studies.

### Biodistribution of mTWNDs

The biodistribution of mTWNDs‐FITC was evaluated in normal and AP mice. Fluorescent drugs were administered, and the pancreas, heart, lungs, spleen, liver, and kidneys were collected at 2, 12, 24, 48, and 72 h following the mTWNDs‐FITC injection. These organs were assessed under a stereofluorescence microscope (Leica, M205FCA) for observation. Each group consisted of four mice (*n =* 4).

### Hematoxylin and Eosin (HE) Staining

The head of the pancreas was treated using the method in section 5.10. Stained by hematoxylin and eosin dyes and then observed under the microscope. Photographs were taken to document the findings. Calculation of HE scores and analysis of the severity of pancreatitis were performed as previously described.^[^ ^10^ ^]^

### Immunohistochemical (IHC) Analysis

The pancreatic tail tissue was embedded and sectioned as described above. Paraffin sections need to be deparaffinized and rehydrated before antigen retrieval using EDTA. Endogenous peroxidase activity was blocked by incubation with an appropriate peroxidase blocker, followed by blocking with bovine serum albumin (5%). Next, the sections were kept overnight at 4 °C with the primary antibodies listed in **Table** **2**. Following washing via PBS, protein levels in tissue were performed by the Histostain‐Plus kit, following the kit protocol. Finally, the results were examined using the histochemical microscope. The specific concentrations and details of each antibody were shown in Table 2.

**Table 2:** Antibody Details of IHC.

Primary/Secondary antibody	Company	Cat. No	Dilution
Cyt c	Proteintech	d10933‐1‐AP	1:500
cGAS	Affinity Bioscience	A8335	1:500
STING	Proteintech	19 851‐1‐AP	1:500
IL‐4	Proteintech	66 142‐1‐Ig	1:500
IL‐1β	Cell Signaling Technology	12242S	1:1000
TNF‐α	Abcam	ab1793	1:1000
IL‐10	Abcam	ab189392	1:1000

### Assessment of Pancreatic Superoxide Production

Pancreatic tissues were collected, stored at −20 °C, and later sectioned into slices via a Cryotome E cryosectioner (Thermo Carlsbad, CA). The sections were stained with DHE (10 µm) for a total of 30 min at 25 °C, and with DAPI for 10 min. The slides were then rinsed three times in PBS, the cover slipped and fixed using an anti‐fluorescence mounting sealer. Finally, the results were analyzed and documented via an orthogonal fluorescence microscope (Nikon ECLIPSE C1).

### In vivo Safety Assessment

Control and mTWNDs groups were included in the safety testing experiments. Mice in the control group were treated with an injection of saline (200 µL), while those in the mTWNDs group were injected with 20 mg k^−1^g of mTWNDs in saline (200 µL). All mice were sacrificed either 1 day or 28 days post‐injection (*n =* 3 per group). Histological changes in various vital organs were evaluated using HE staining, Masson staining, and Congo red staining to analyze the toxicity of the mTWNDs. Blood samples were collected for analysis of LYM, WBC, HGB, RBC, MON, and PLT. Hepatic function indicators, including ALT and AST, as well as renal function parameters, including BUN and SCr, were assessed using the Chemray 240 automated biochemistry analyzer.

### Isolation of PPACs

PPACs were isolated by collagenase digestion of mouse pancreatic tissue using a modified version of a previously described protocol.^[^ ^10^ ^]^ Fresh pancreatic tissue was injected with Buffer A, consisting of soybean trypsin inhibitor (pH 7.3), CaCl_2_ (1 mm), MgCl_2_ (1.13 mm), glucose (10 mm), KCl (4.7 mm), HEPES (10 mm), and NaCl (140 mm), supplemented with 1 mg mL^−1^ collagenase (IV). The tissue was sheared and digested at 37 °C while shaking at 100 rpm ≈15 min. Finally, the digested mixture was then passed using a 100‐µm nylon mesh filter to collect scattered PPACs.

### Cells Culture

Macrophages‐RAW 264.7 were obtained from the National Center for the Conservation of Authenticated Cell Cultures, China. The medium for PPACs and RAW 264.7 was DMEM/F12 supplemented with 10% FBS. Incubation conditions included humidified conditions at 37 °C and 5% CO_2_. DMEM Dulcimer's Modified Eagle's Medium was abbreviated as DMEM and fetal bovine serum was abbreviated as FBS.

### Establishment of in vitro Cellular Models

PPACs were categorized into three groups: normal control, AP model, as well as mTWNDs‐treated groups. The normal control group was cultured in a standard medium, while the AP model and drug‐treated groups were induced with 10 µm CCK for 6 h. For the drug‐treated group, mTWNDs were introduced into the culture medium and incubated for an additional 12 h. Subsequently, cells and supernatants were collected for further analysis. Supernatants from each group of PPACs were then used to culture macrophages. Macrophages were grown in the PPACs supernatants for 12 h, after which the medium was substituted with fresh culture medium for an additional 12‐hour incubation. Finally, macrophages and their supernatants were collected for subsequent studies.

### Mitochondrial Function

PPACs were treated as described above, stained following the instructions provided in the MitoSOX kit, and subsequently analyzed using flow cytometry (01‐DXPSF13‐01, Cytek, DxpAthena) or fluorescence microscopy.

MMP was measured using the JC‐1 kit. PPACs were treated as described above, stained according to the kit instructions, and analyzed using flow cytometry and fluorescence microscopy.

### Apoptosis Analysis

Apoptosis analysis of pancreatic tissues was conducted using TUNEL staining, executed following the kit protocol. Annexin V FITC analysis was utilized to assess the apoptosis of PPACs. PPACs were first collected by trypsinization and rinsed twice with pre‐cooled FBS. Next, centrifugation and resuspension resulted in 1 × 106 cells mL^−1^. Subsequently, single‐cell suspensions were transferred to new tubes for Annexin V FITC and propidium iodide (PI) staining. Protect the mixed liquid from light ≈15 min at 25 °C. Following adding the binding buffer, the results were analyzed using flow cytometry (Cytek, DxpAthena, 01‐DXPSF13‐01).

### Subcellular Localization

The localization of mTWNDs‐FITC in cells was observed using fluorescence microscopy. PPACs were cultured into 24‐well plates at 5 × 10^4^ cells/well, and incubated with mTWNDs‐FITC for 12 h. Mitochondria were labeled using Mito‐Tracker Red CMXRos. Results were acquired with a Zeiss fluorescence microscope (LSM 900, Germany).

### ATP Assays

ATP production in PPACs was measured utilizing an ATP detection kit. Cells were treated as described in Section 5.18, and the detection was conducted following the kit protocol.

### Immunofluorescence Staining

Dewax and hydrate the paraffin sections, perform antigen retrieval with ethylenediaminetetraacetic acid and permeabilize with PBS containing Triton X‐100 (0.1%), followed by blocking with BSA‐PBS (5%). Samples were kept overnight at 4 °C along with primary antibodies targeting F4/80, dsDNA + TOM20, or STING + CD68. Following washing, slides were incubated in the dark with corresponding specialized secondary antibodies (Alexa Fluor 488 or Alexa Fluor 555) diluted in 1% BSA. Finally, a DAPI solution was utilized to dye the nuclei, and images were captured via a special microscope.

RAW 264.7 cells and PPACs were seeded on coverslips, washed twice using PBS, and then immobilized in paraformaldehyde (4%). Cellular immunofluorescence experiments were conducted following the same method. Primary antibodies used in cellular immunofluorescence included COX‐2, iNOS, and dsDNA + TOM20. The specific concentrations and details of each antibody were shown in **Table** **3**.

**Table 3:** Antibody Details of Immunofluorescence Staining.

Primary/Secondary antibody	Company	Cat. No	Dilution
Anti‐F4/80 antibody	Abcam	AB6640	1:500
Anti‐dsDNA antibody	Abcam	AB27156	1:500
TOM20 Polyclonal antibody	Proteintech	11 802‐1‐AP	1:500
COX‐2 antibody	Proteintech	27 308‐1‐4P	1:500
CD68 Recombinant antibody	Proteintech	28 058‐1‐AP	1:100
iNOS antibody	Affinity Bioscience	AF0199	1:200
Alexa Fluor™ 555	Invitrogen	A21428	1:500
Alexa Fluor™ 488	Invitrogen	A11029	1:500

### Cytotoxicity Assay

PPACs were grown into 96‐well plates and administered with different doses of mTWNDs ≈24 h. After treatment, the culture fluid was substituted with 10% CCK‐8 medium, and PPACs were cultured at 37 °C ≈30 min. A microplate reader was utilized to determine absorbance at 450 nm.

### Western Blotting

All protein extraction from pancreatic tissues or cells was performed using RIPA buffer containing phosphatase inhibitors and phenylmethanesulfonyl fluoride. The supernatant was obtained by high‐speed centrifugation at 4 °C and protein level was analyzed with the Bicinchoninic Acid Assay Kit. Next, the proteins were sequentially separated by electrophoresis, as well as transferred to the PDVF membrane. This was followed by sealing with a special sealing solution. Next, the resulting membranes were kept refrigerated overnight with primary antibodies at the concentrations specified in **Table** **4**. Bound antibodies were detected with appropriate secondary antibodies and chemiluminescent visualization was performed. Protein bands were processed using ImageJ software.

**Table 4:** Antibody list of Western Blotting.

Primary/Secondary antibody	Company	Cat. No	Dilution
GAPDH	Cell Signaling Technology	97166S	1:2000
Cyt c	Proteintech	d10933‐1‐AP	1:1000
BAX	Abcam	AB32503	1:1000
BCL‐2	Affinity Bioscience	BF9103	1:1000
cGAS	Affinity Bioscience	A8335	1:1000
STING	Proteintech	19 851‐1‐AP	1:1000
p‐IRF3	Affinity Bioscience	AF2436	1:1000
IRF3	Affinity Bioscience	DF6895	1:1000
p‐P65	Affinity Bioscience	AF2006	1:1000
P65	Affinity Bioscience	BF8005	1:1000
cGAS	Proteintech	29 958‐1‐AP	1:1000
STING	Proteintech	19 851‐1‐AP	1:1000
COX‐2	Proteintech	27 308‐1‐4P	1:1000
iNOS	Affinity Bioscience	AF0199	1:1000
Goat anti‐Rabbit IgG H&L (HRP)	Abclonal	AS014	1:4000
Goat anti‐Mouse IgG H&L (HRP)	Abclonal	AS003	1:4000

### Inflammatory Factor Detection

A 10% homogenate of pancreatic tissue or PPACs was obtained. Levels of various inflammatory factors in pancreatic tissue or PPACs were determined utilizing ELISA kits, according to the kit protocol. The details of the ELISA kits used were shown in **Table** **5**.

**Table 5:** Details of Elisa kit for Mouse.

Primary/Secondary antibody	Company	Cat. No
Mouse IL‐1β ELISA Kit	Elabscience	E‐EL‐M0037
Mouse TNF‐α ELISA Kit	Elabscience	E‐EL‐M3063
Mouse IL‐4 ELISA Kit	Elabscience	E‐EL‐M0043
Mouse IL‐10 ELISA Kit	Elabscience	E‐EL‐M0046

### Statistical Analysis

All data in this study were mean ± S.D. and were from at least three independent trials. All data were processed utilizing GraphPad Prism software version 8.0 (USA), SPSS 22.0 (SPSS Inc., USA), ImageJ (version 1.8.0), and Origin (2024). An unpaired t‐test was conducted for the statistical analysis in Figures 1 and 6, while one‐way ANOVA was conducted on the remaining figures to establish the statistical differences. A p‐value below 0.05 was deemed to be statistically significant.

## Conflict of Interest

The authors declare no conflict of interest.

## Author Contributions

D.W. and S.W. contributed equally to the work. K.A., X.G., and Q.H. conceived the concept. S.W. and X.S. designed and synthesized all the nanomedicines used in this study. D.W. and J.L. performed the experiments. D.W. wrote the paper. D.W., S.W., X.S., L.X., R.L., W.W., L.J., Q.H., X.G., and K.A. reviewed and commented on the paper.

## Supporting information



Supporting Information

## Data Availability

The article and its supplementary information contain all data processed in this study. Additional raw data can be provided upon request from the corresponding author.
