# Supplementary material for: Nanomedicine Penetrating Blood‐Pancreas Barrier for Effective Treatment of Acute Pancreatitis
Source: Adv Sci (Weinh). 2025 Feb 14;12(13):2413925. doi: 10.1002/advs.202413925 (PMC11967758; doi:10.1002/advs.202413925)
Supplement: Supplementary file 1 — Supporting Information [file ADVS-12-2413925-s001.docx]

Supporting Information

**Nanomedicine Penetrating Blood-Pancreas Barrier for Effective Treatment of Acute Pancreatitis**

Dan Wang^1,4†^, Shuya Wang^2†^, Jinjin Liu^1,4^, Xiaojing Shi^2^, Tingli Xiong^2^, Ruishi Li^2^, Wei Wei^1,4^, Liandong Ji^1,4^, Qiong Huang^3,4*^, Xuejun Gong^1,4*^, Kelong Ai^2,5,6*^

^1^Department of General Surgery, Xiangya Hospital, Central South University, Changsha, 410008, China.

^2^Xiangya School of Pharmaceutical Sciences, Central South University, Changsha, 410013, China.

^3^Department of Pharmacy, Xiangya Hospital, Central South University, Changsha, 410008, China.

^4^National Clinical Research Center for Geriatric Disorders, Xiangya Hospital, Central South University, Changsha, 410008, China.

^5^Hunan Provincial Key Laboratory of Cardiovascular Research, Xiangya School of Pharmaceutical Sciences, Central South University, Changsha, 410013, China.

^6^Key Laboratory of Aging-related Bone and Joint Diseases Prevention and Treatment, Ministry of Education, Xiangya Hospital, Central South University, Changsha, 410008, China.

^†^ The authors Dan Wang and Shuya Wang contributed equally to the work.

^*^Corresponding authors: qionghuang@csu.edu.cn (Prof. Qiong Huang), [peigong158@csu.edu.cn](mailto:peigong158@csu.edu.cn) (Prof. Xuejun Gong), aikelong@csu.edu.cn (Prof. Kelong Ai).


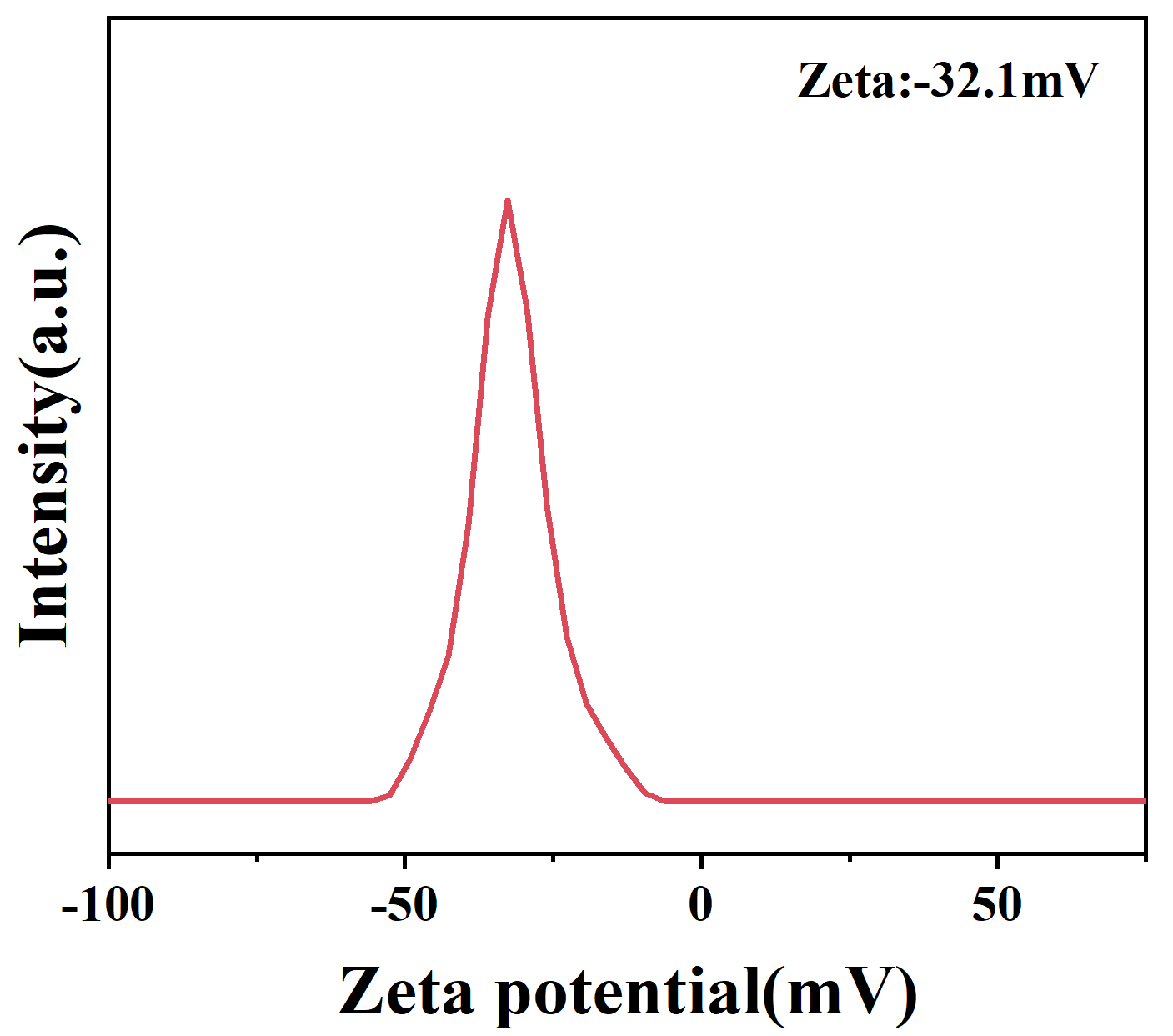


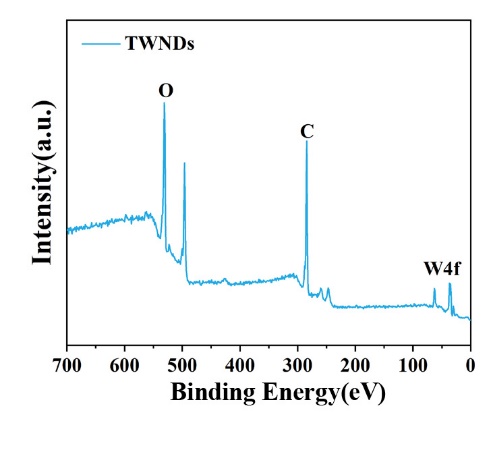
**Figure S1**. Zeta potential determination of mTWNDs.

**Figure S2**. Total spectrum energy of TWNDs.


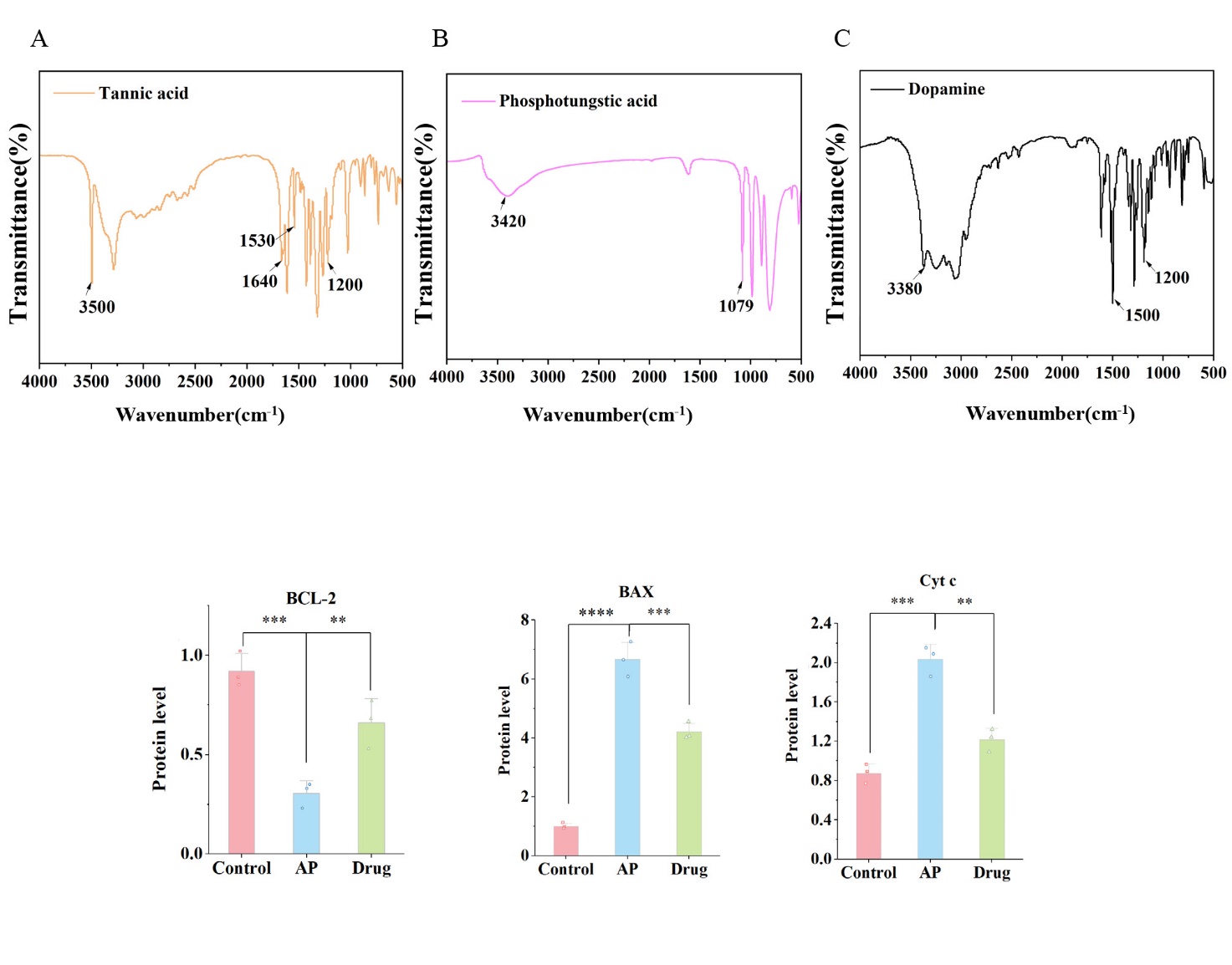


**Figure S3**. FTIR spectrum of Tannic acid, Phosphotungstic acid and Dopamine. A. FTIR spectrum of Tannic acid shows characteristic absorption peaks of hydroxyl, carbonyl, benzene ring, and phenolic hydroxyl can be detected at 3500 cm^-1^, 1640 cm^-1^, 1530 cm^-1^ and 1200 cm^-1^. B. FTIR spectrum of Phosphotungstic acid exhibits characteristic absorption peaks of hydroxyl and molybdenum can be detected at 3420 cm^-1^, 1079 cm^-1^. C. FTIR spectrum of Dopamine demonstrates characteristic absorption peaks of hydroxyl, benzene ring and phenolic hydroxyl can be detected at 3380 cm^-1^, 1500 cm^-1^ and 1200 cm^-1^.





**Figure S4**. UV-Vis-NIR spectrum of mTWNDs, Tannic acid, Dopamine, and Phosphotungstic acid.


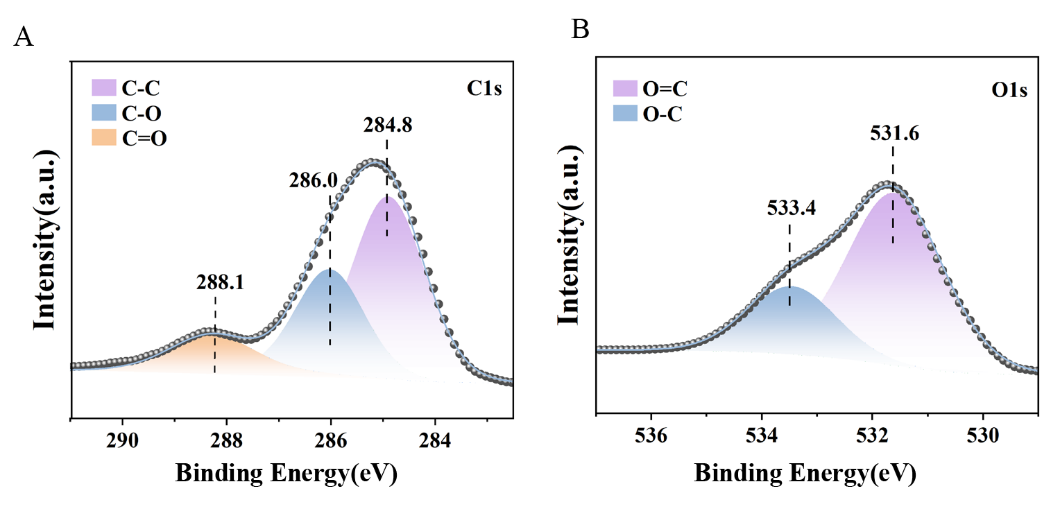


**Figure S5**. XPS spectrum of mTWNDs. A. C1s narrow scan XPS spectrum. B. O1s narrow scan XPS spectrum.


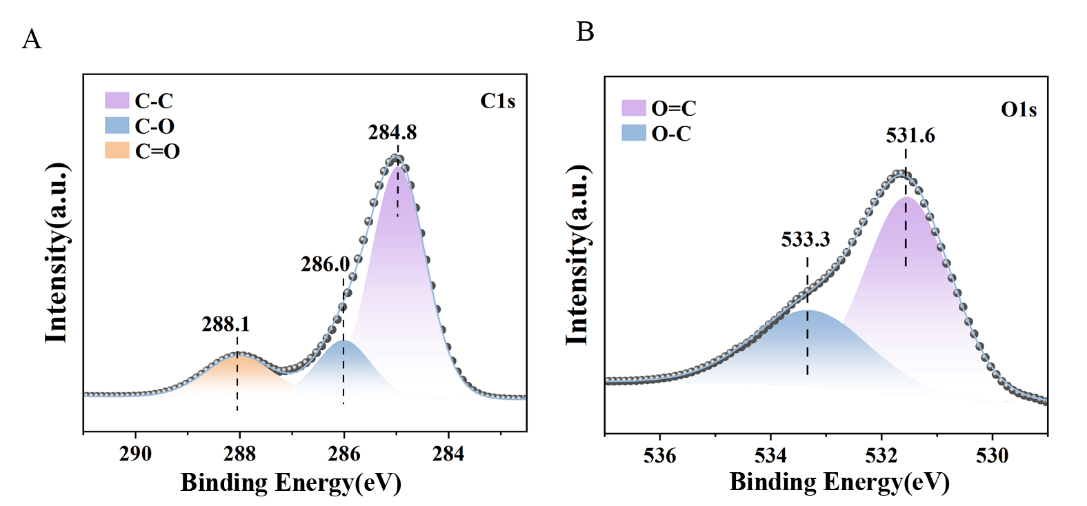


**Figure S6**. XPS spectrum of TWNDs. A. C1s narrow scan XPS spectrum. B. O1s narrow scan XPS spectrum.


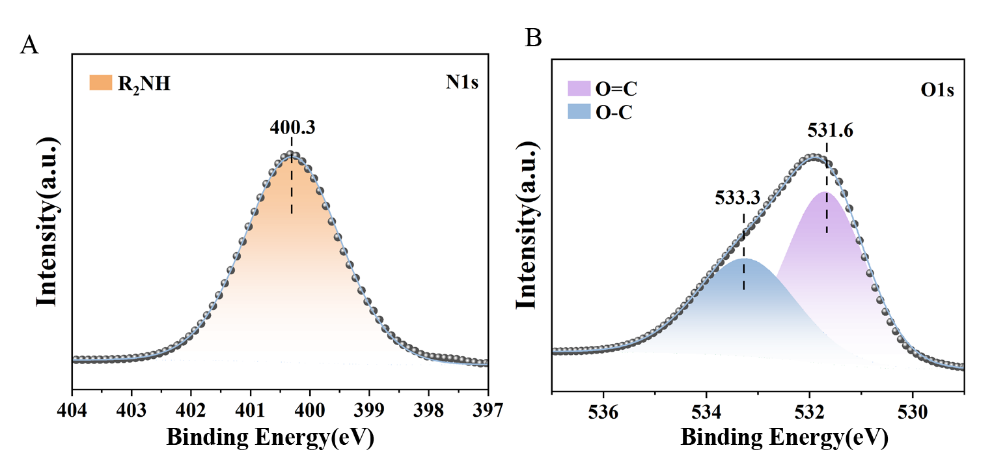


**Figure S7**. XPS spectrum of mTWNDs after reaction with H_2_O_2_. A. N1s narrow scan XPS spectrum. B. O1s narrow scan XPS spectrum.


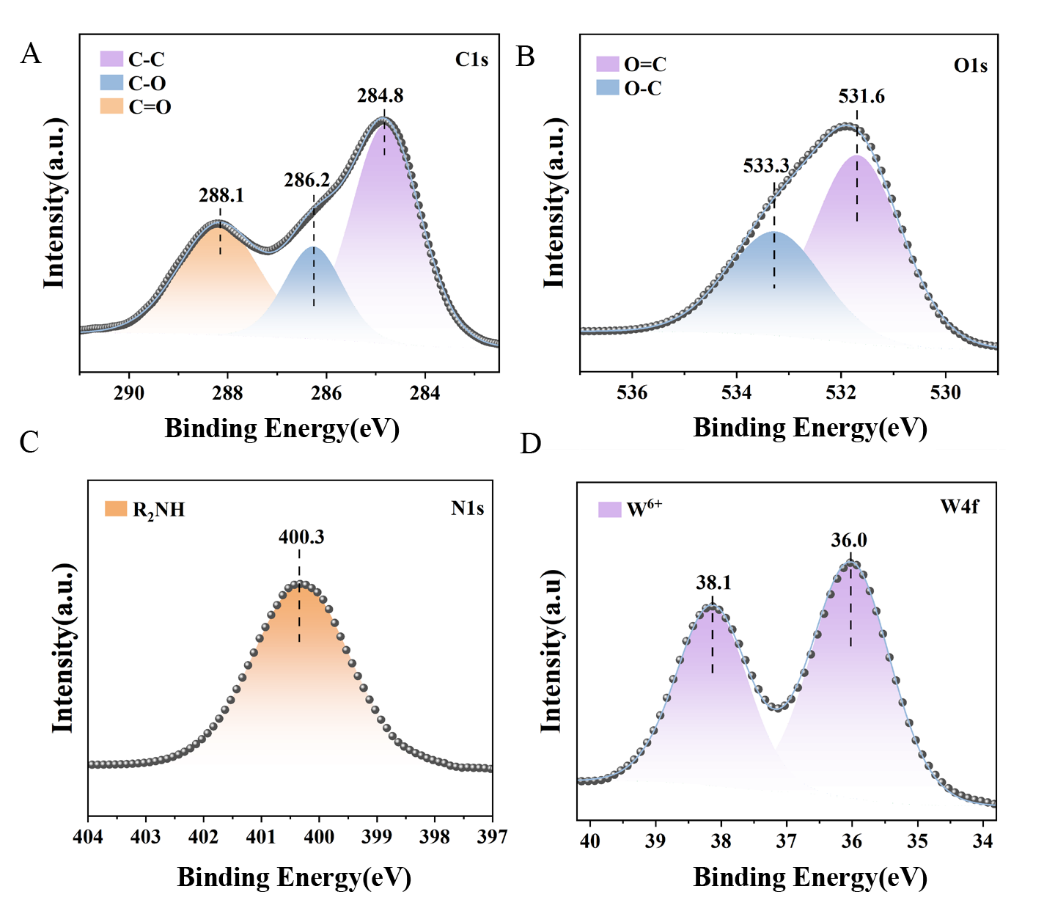


**Figure S8**. XPS spectrum of mTWNDs after reaction with ·OH. A. C1s narrow scan XPS spectrum. B. O1s narrow scan XPS spectrum. C. N1s narrow scan XPS spectrum. D. W4f narrow scan XPS spectrum.


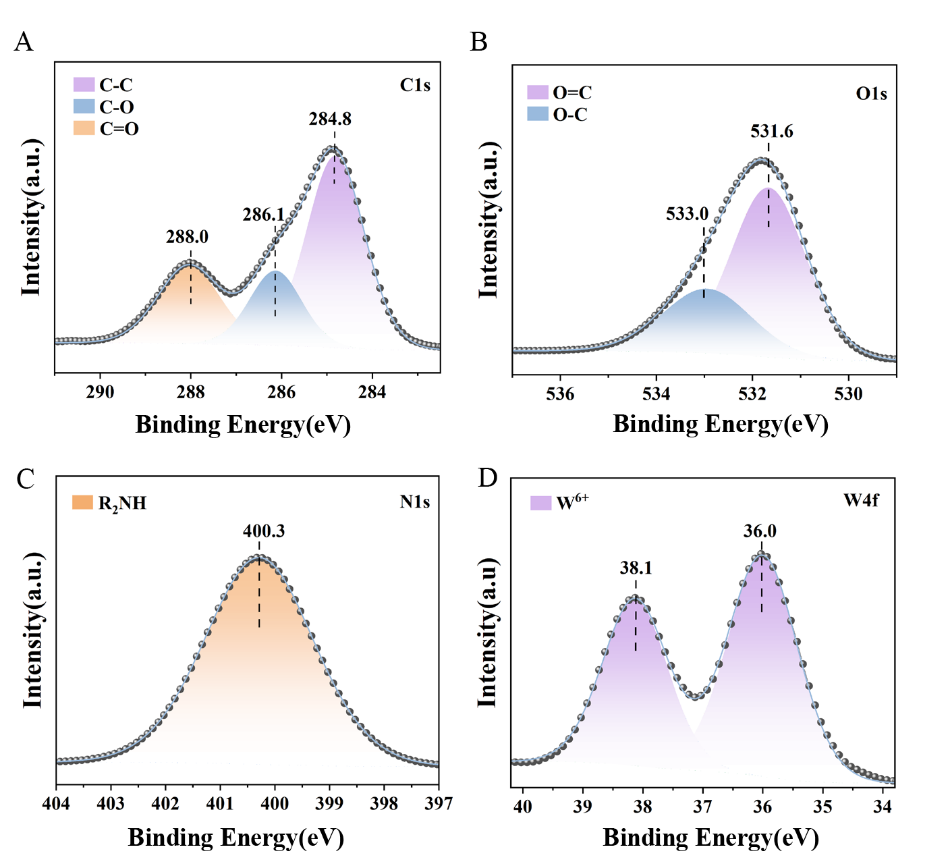


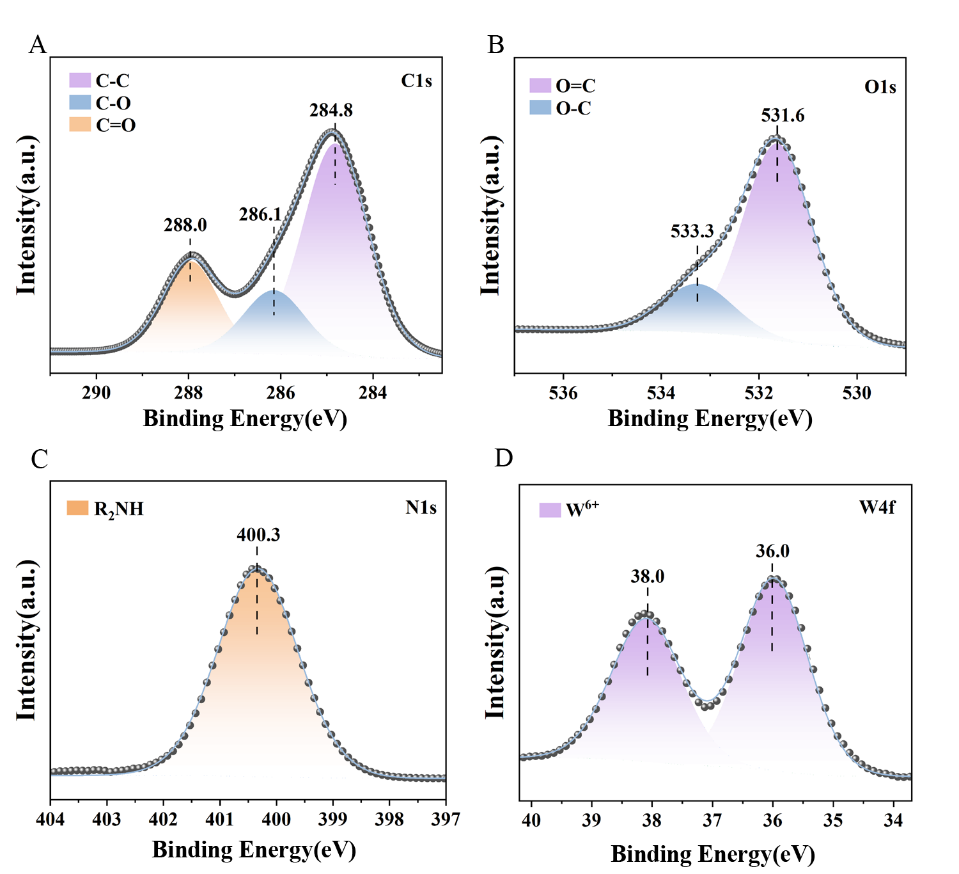
**Figure S9**. XPS spectrum of mTWNDs after reaction with O_2_^•-^. A. C1s narrow scan XPS spectrum. B. O1s narrow scan XPS spectrum. C. N1s narrow scan XPS spectrum. D. W4f narrow scan XPS spectrum.

**Figure S10**. XPS spectrum of mTWNDs after reaction with ONOO^-^. A. C1s narrow scan XPS spectrum. B. O1s narrow scan XPS spectrum. C. N1s narrow scan XPS spectrum. D. W4f narrow scan XPS spectrum.


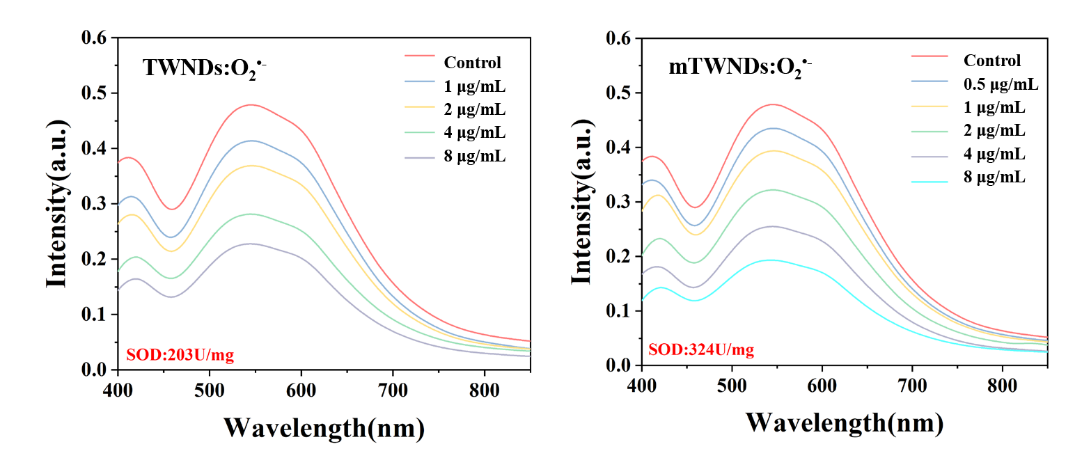


**Figure S11**. In vitro O_2_^•-^ scavenging ability of TWNDs and mTWNDs.


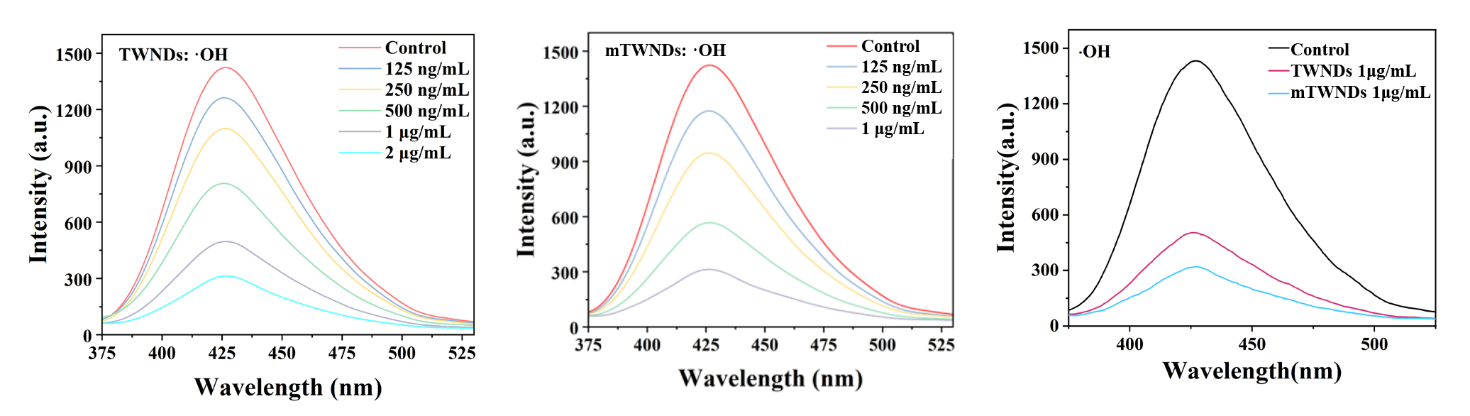


**Figure S12**. In vitro ·OH scavenging ability of TWNDs and mTWNDs.


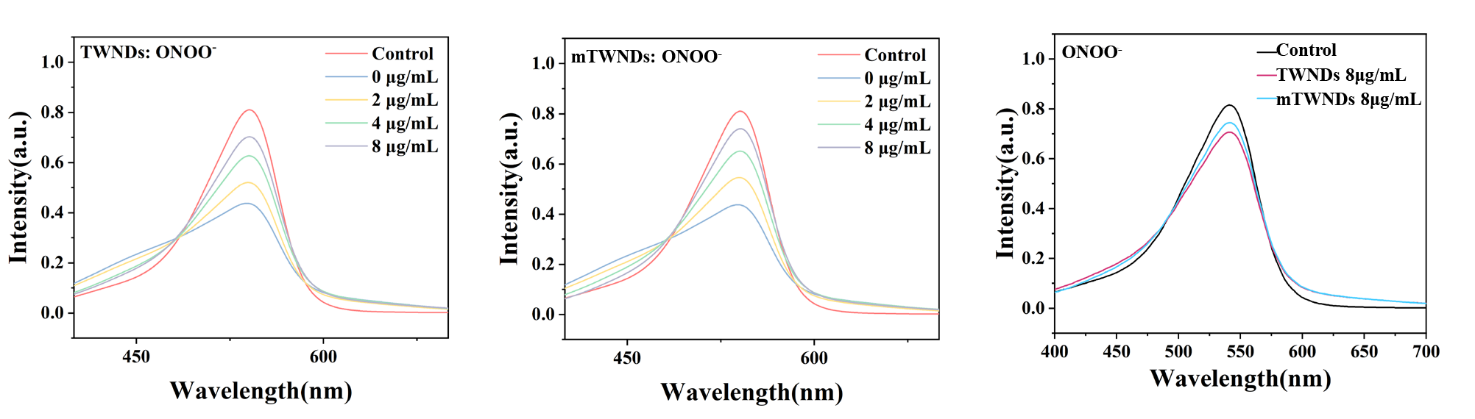


**Figure S13**. In vitro ONOO^-^ scavenging ability of TWNDs and mTWNDs.


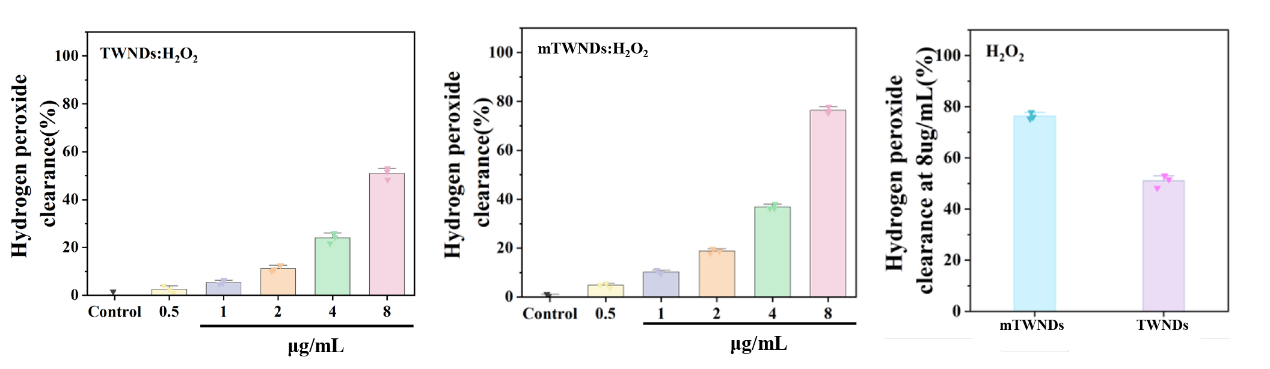


**Figure S14**. In vitro H_2_O_2_ scavenging ability of TWNDs and mTWNDs. Data represent mean ± S.D. (n = 3 independent experiments).





**Figure S15.** Vis-NIR spectrum change of mTWNDs under oxidative stress (O_2_^.-^, ONOO^-^, ·OH, H_2_O_2_) systems. The aqueous solution of mTWNDs serves as the control.


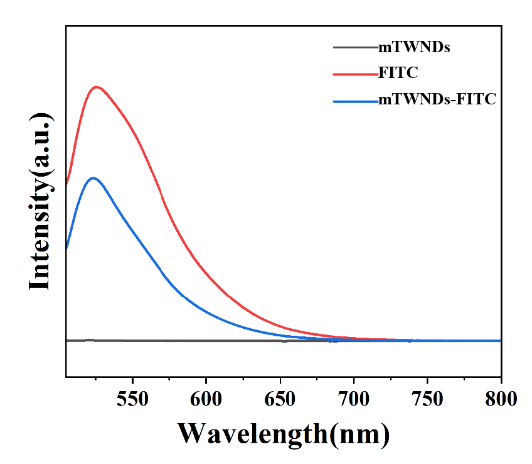


**Figure S16.** UV**-**Vis-NIR spectrum of mTWNDs, FITC and mTWNDs-FITC.


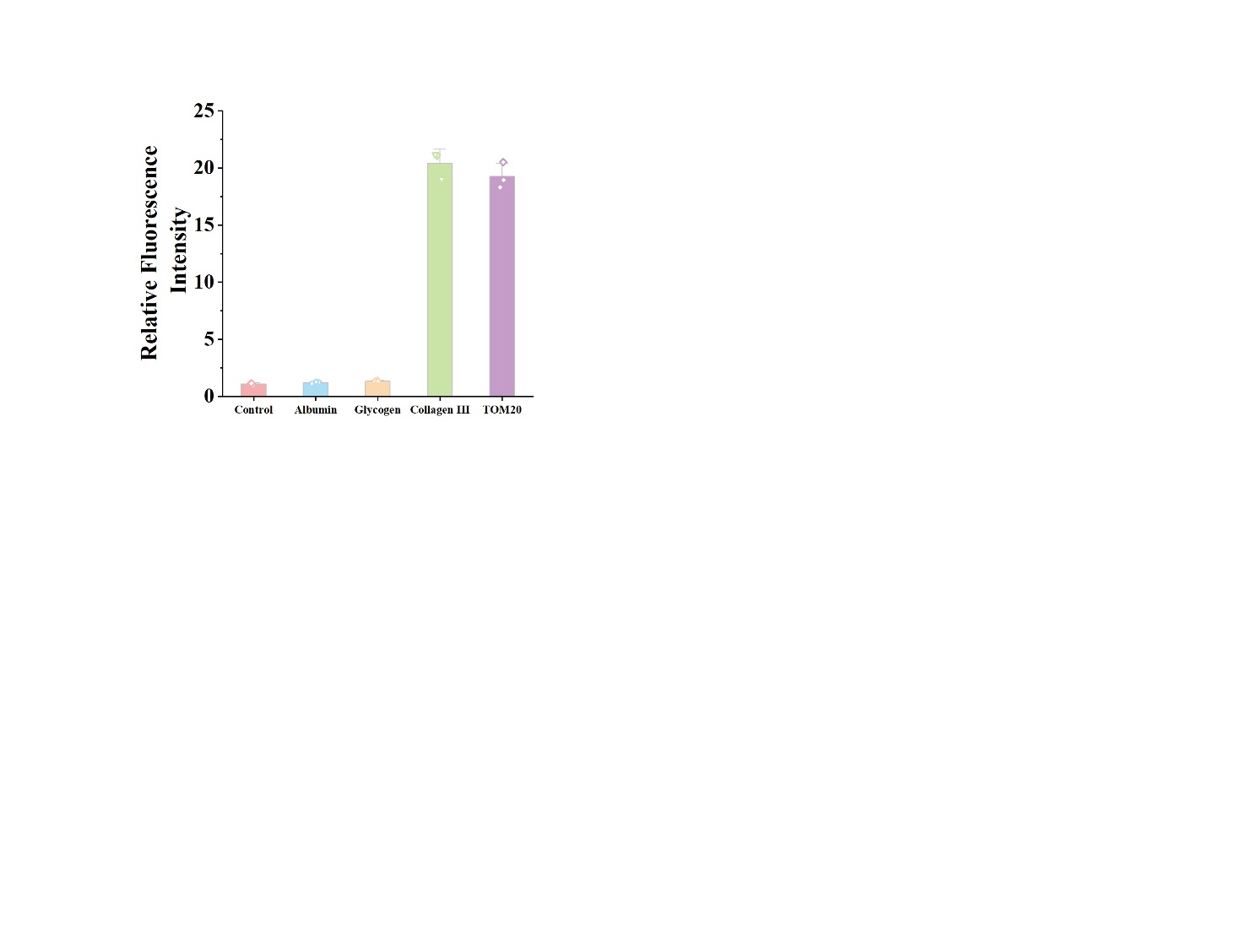


**Figure S17**. Quantitative analysis of the protein-coated plate experiment demonstrated that mTWNDs could bind to collagen III and TOM20. Data represent mean ± S.D. (n = 3 independent experiments).


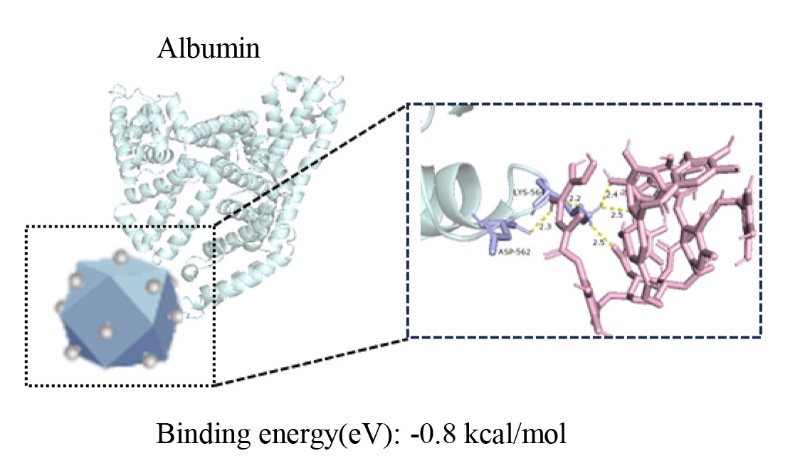


**Figure S18.** Results of molecular docking of Tannic acid with Albumin protein.


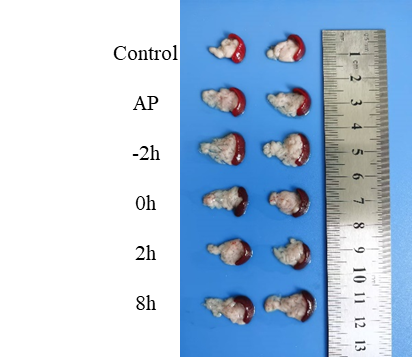





**Figure S19.** Physical images of mouse pancreatic organs at different time points and pancreas-to-body weight ratio (WP/BW). Data represent mean ± S.D. Statistical signiﬁcance was determined using one-way ANOVA. (n = 4 animals per group, ^**^ for *P* < 0.01 vs. AP group).





**Figure S20.** Serum levels of AMY at different time points. Data represent mean ± S.D. Statistical signiﬁcance was determined using one-way ANOVA. (n = 3 animals per group, ^**^ for *P* < 0.01 vs. AP group).

A


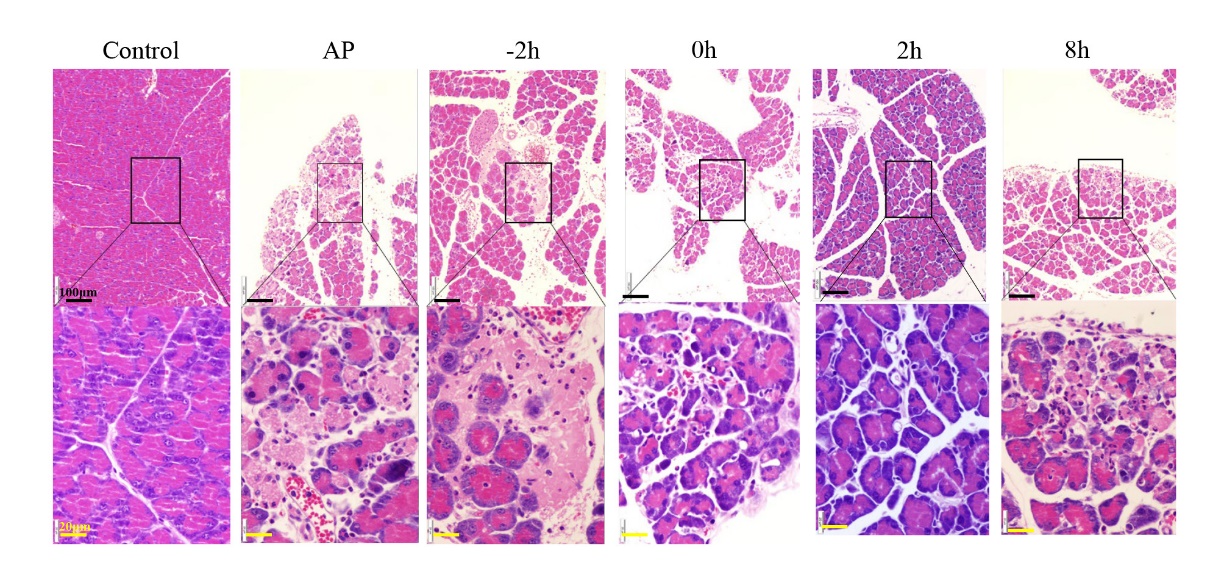









D

C

B

**Figure S21.** A. HE staining of pancreas tissues from each group. B-D. Edema (B), acinar necrosis (C), and inflammatory infiltrate (D) scores for each group in HE staining. Data represent mean ± S.D. Statistical signiﬁcance was determined using one-way ANOVA. (n = 3 animals per group, ^*^ for *P* < 0.05, ^**^ for *P* < 0.01 vs. AP group).


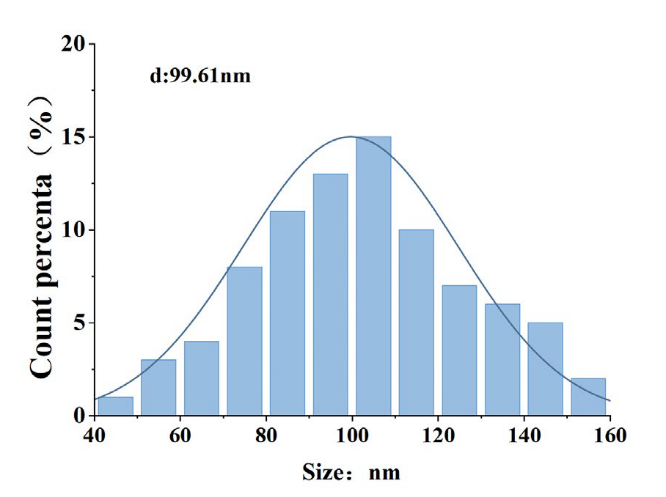


**Figure S22.** Histogram of the size of endothelial cell gaps in pancreatic tissue of AP.


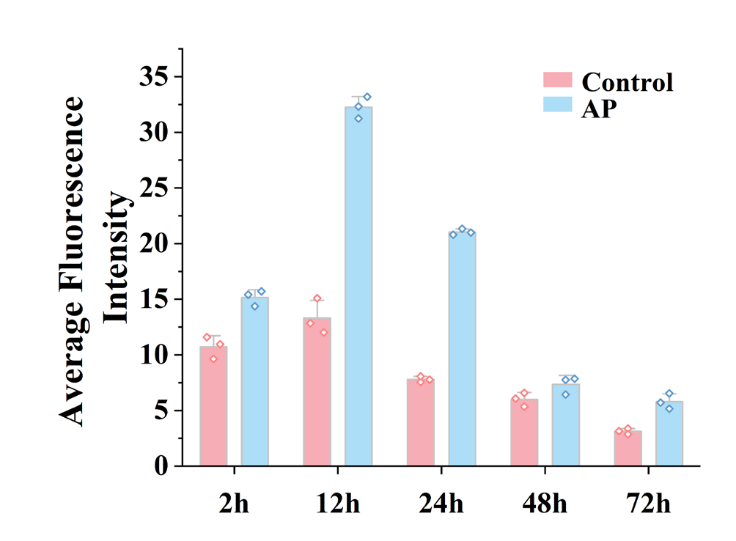


**Figure S23.** Quantitative analysis of the stereoscopic fluorescence distribution of mTWNDs in the pancreas at different time points. Data represent mean ± S.D. (n = 3 animals per group).


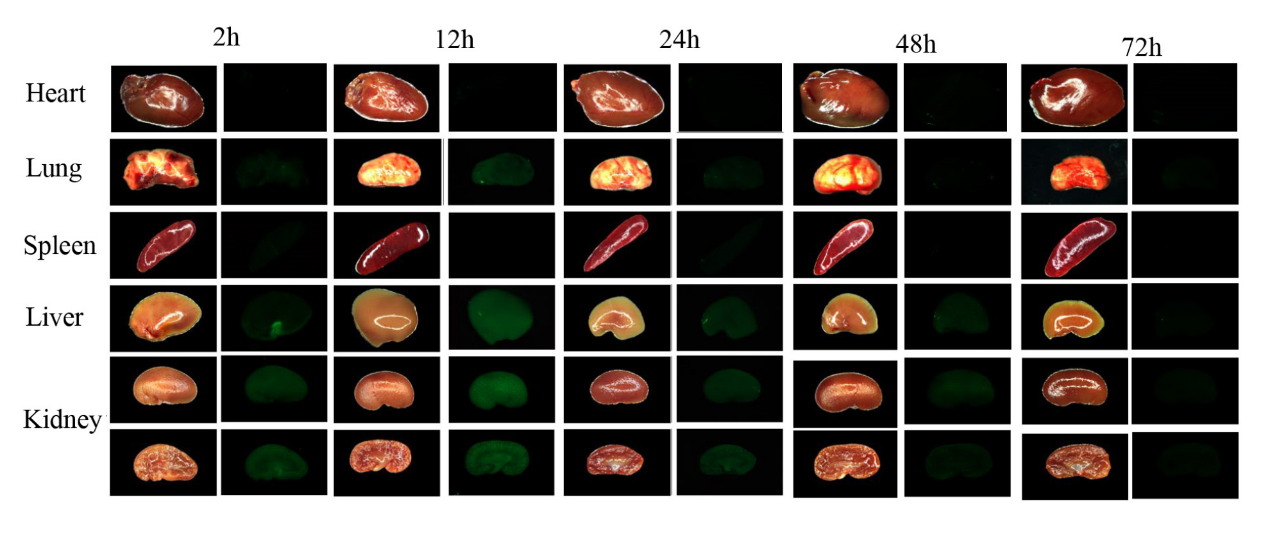


**Figure S24.** Control group: In vivo distribution of stereofluorescence of mTWNDs at different times.


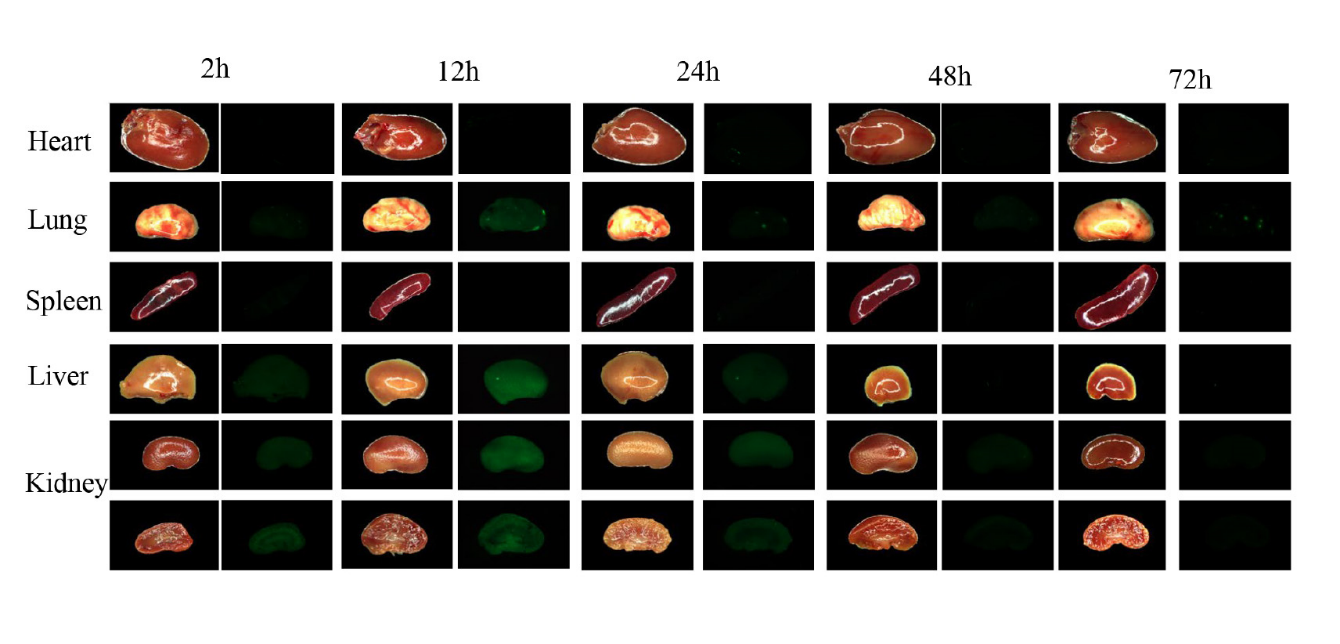


**Figure S25.** AP group: In vivo distribution of stereofluorescence of mTWNDs at different times.

**
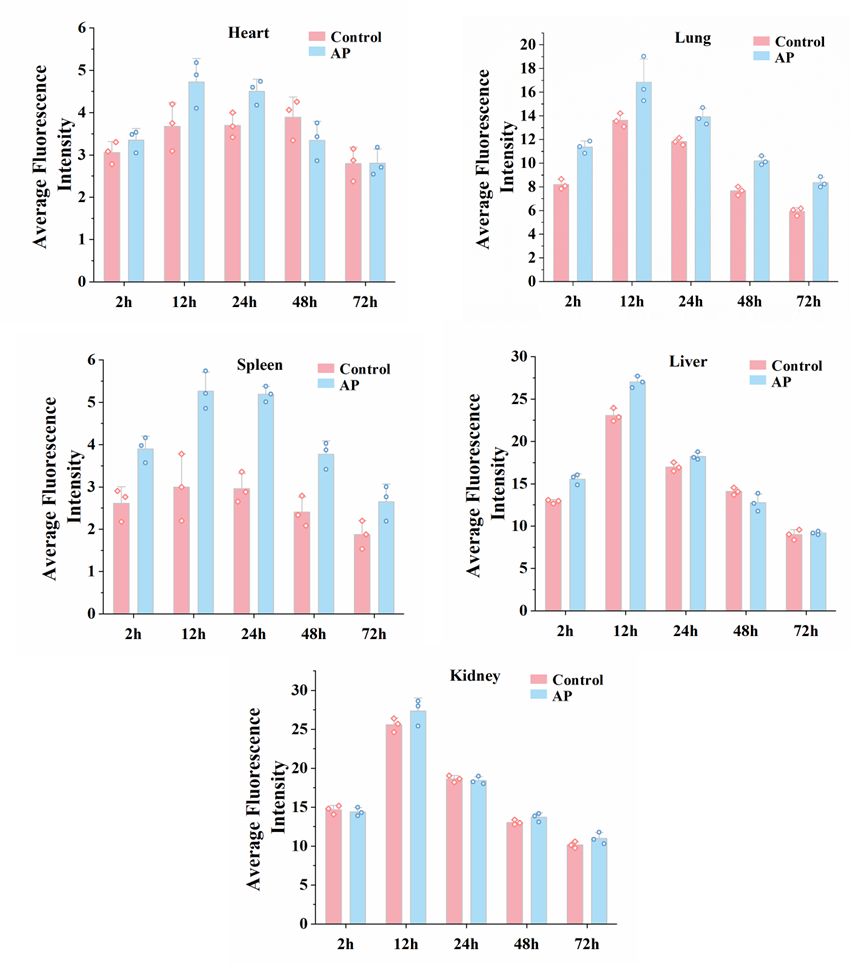
**

**Figure S26.** Quantitative statistics of in vivo distribution of fluorescence intensity in different organs. Data represent mean ± S.D. (n = 3 animals per group).


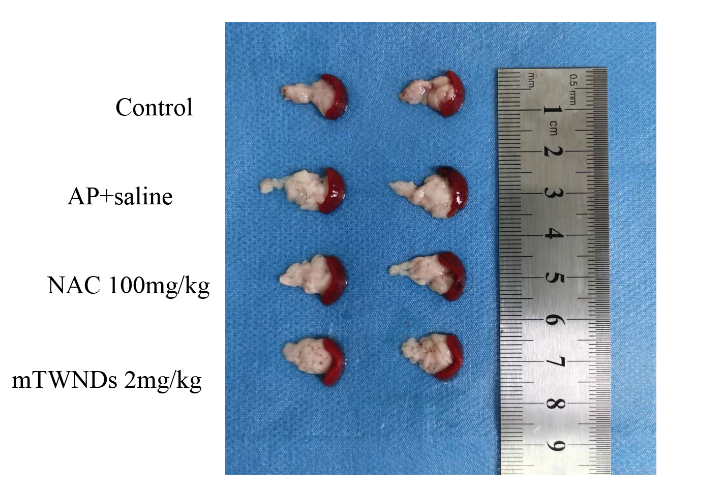


**Figure S27.** Physical images of mouse pancreatic organs in different groups.


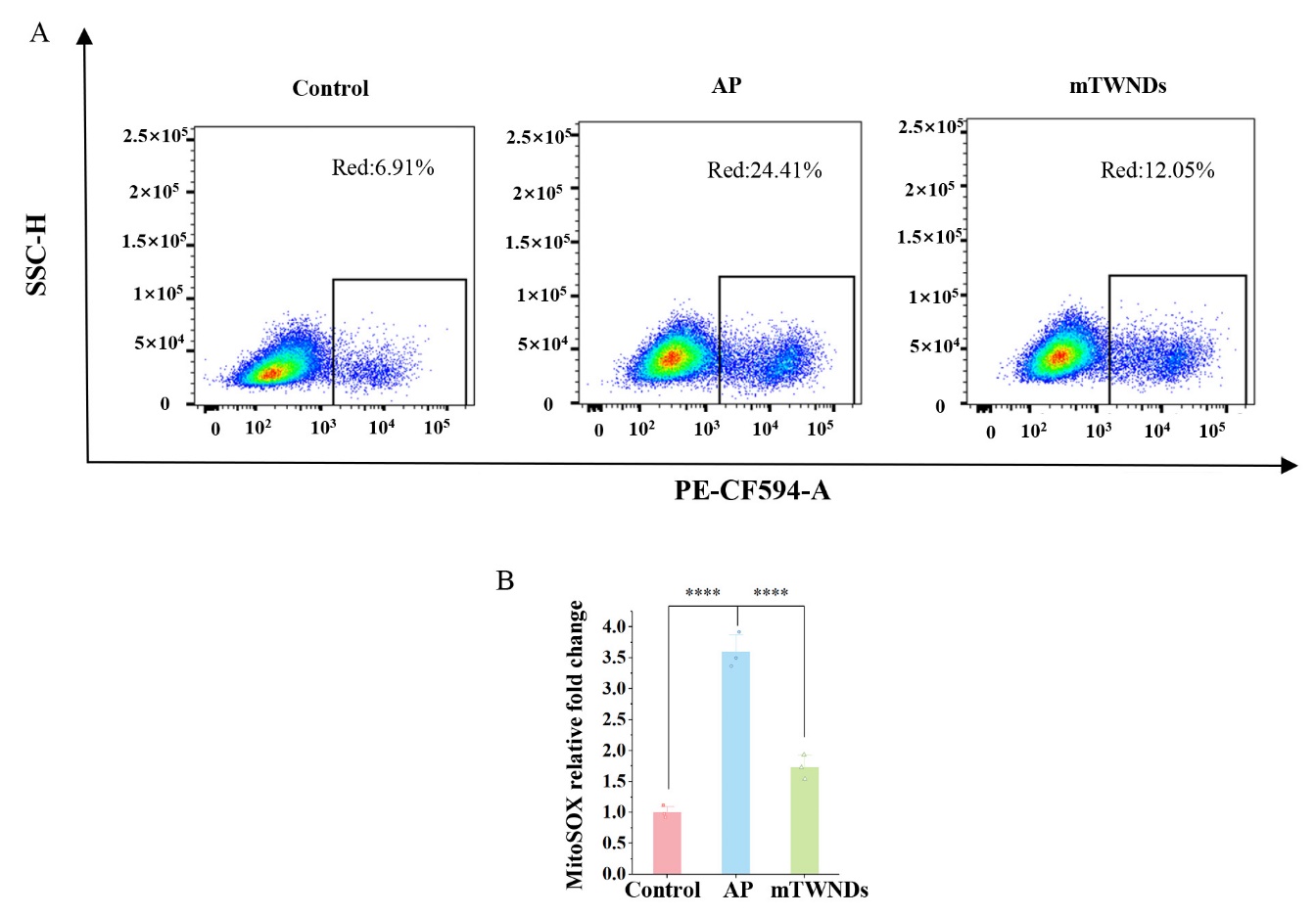


**Figure S28.** MitoSOX flow cytometry results of PPACs under different treatment conditions (A) and quantitative statistical results of MitoSOX fluorescence intensity (B). Data represent mean ± S.D. Statistical signiﬁcance was determined using one-way ANOVA. (n = 3 independent experiments, ^****^ for *P* < 0.0001 vs. AP group).


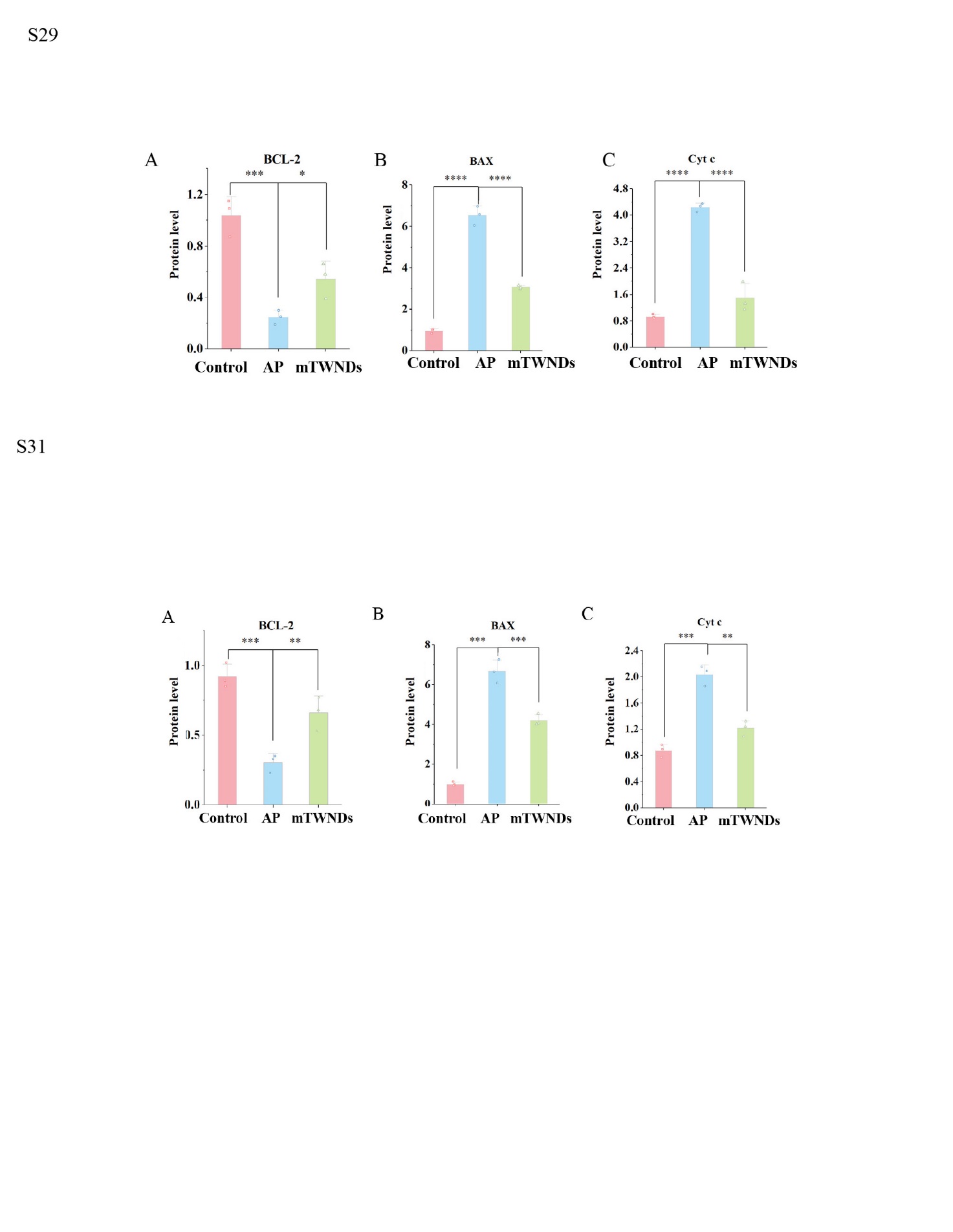


**Figure S29.** Quantification of the protein immunoblots of BAX, BCL-2, and Cyt c. Data represent mean ± S.D. Statistical signiﬁcance was determined using one-way ANOVA. (n = 3 independent experiments, ^*^ for *P* < 0.05, ^**^ for *P* < 0.01, ^***^ for *P* < 0.001, ^****^ for *P* < 0.0001 vs. AP group).


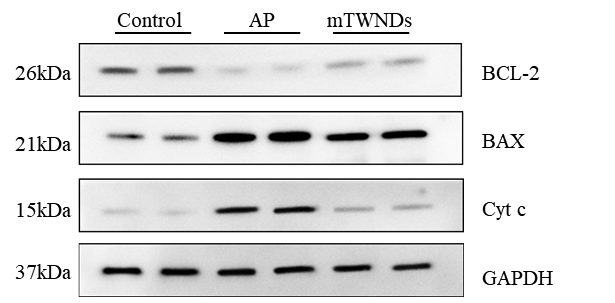


**Figure S30.** Western blot analysis of BAX, BCL-2, and Cyt c proteins in pancreatic tissue from each group.


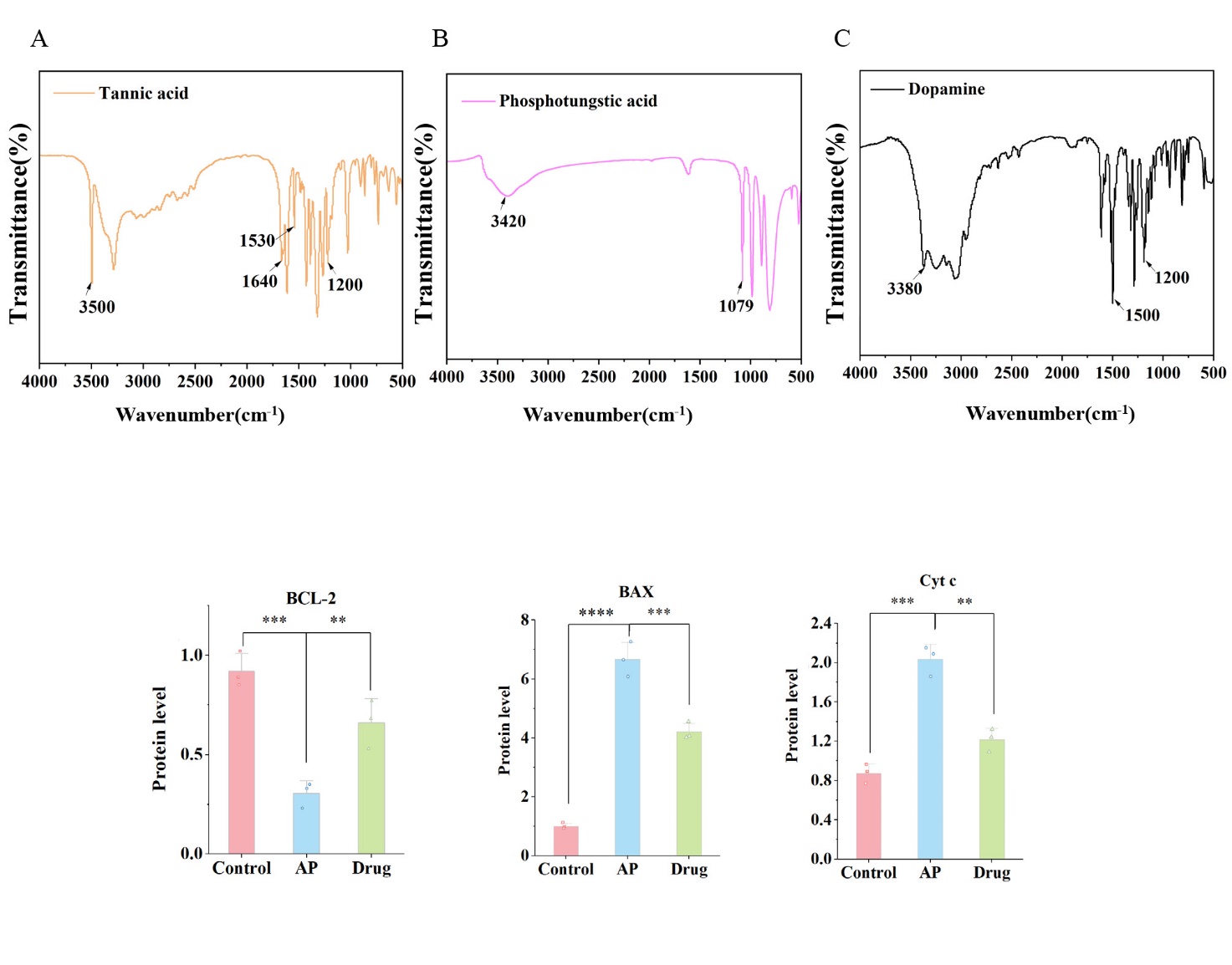


**Figure S31.** Quantification of the protein immunoblots of BAX, BCL-2, and Cyt c. Data represent mean ± S.D. Statistical signiﬁcance was determined using one-way ANOVA. (n = 3 animals per group, ^*^ for *P* < 0.05, ^**^ for *P* < 0.01, ^***^ for *P* < 0.001, ^****^ for *P* < 0.0001 vs. AP group).


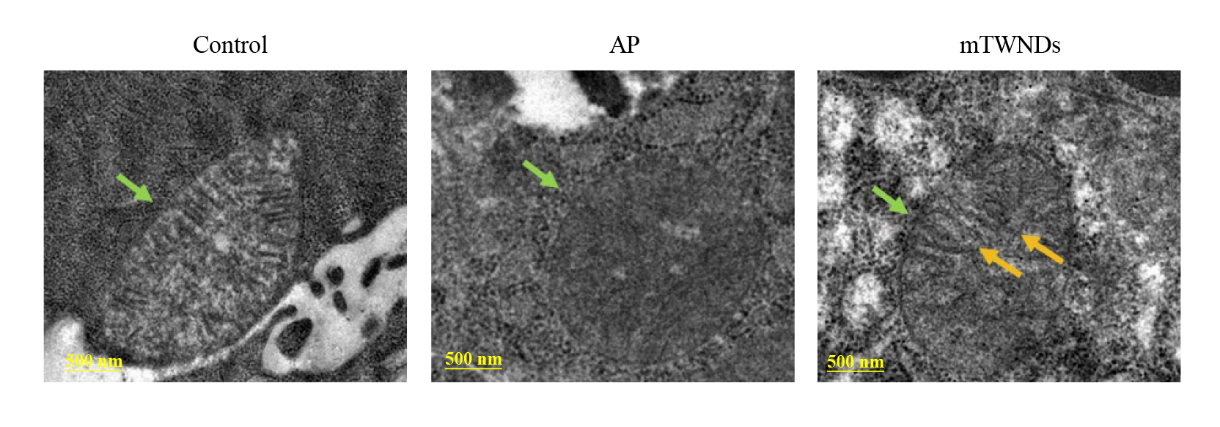


**Figure S32.** TEM image of mitochondria (indicated by the green arrow) in pancreatic tissue from the Control group, AP group, and mTWNDs treatment group. The yellow arrows point to mTWNDs. Scale bar: 500 nm.


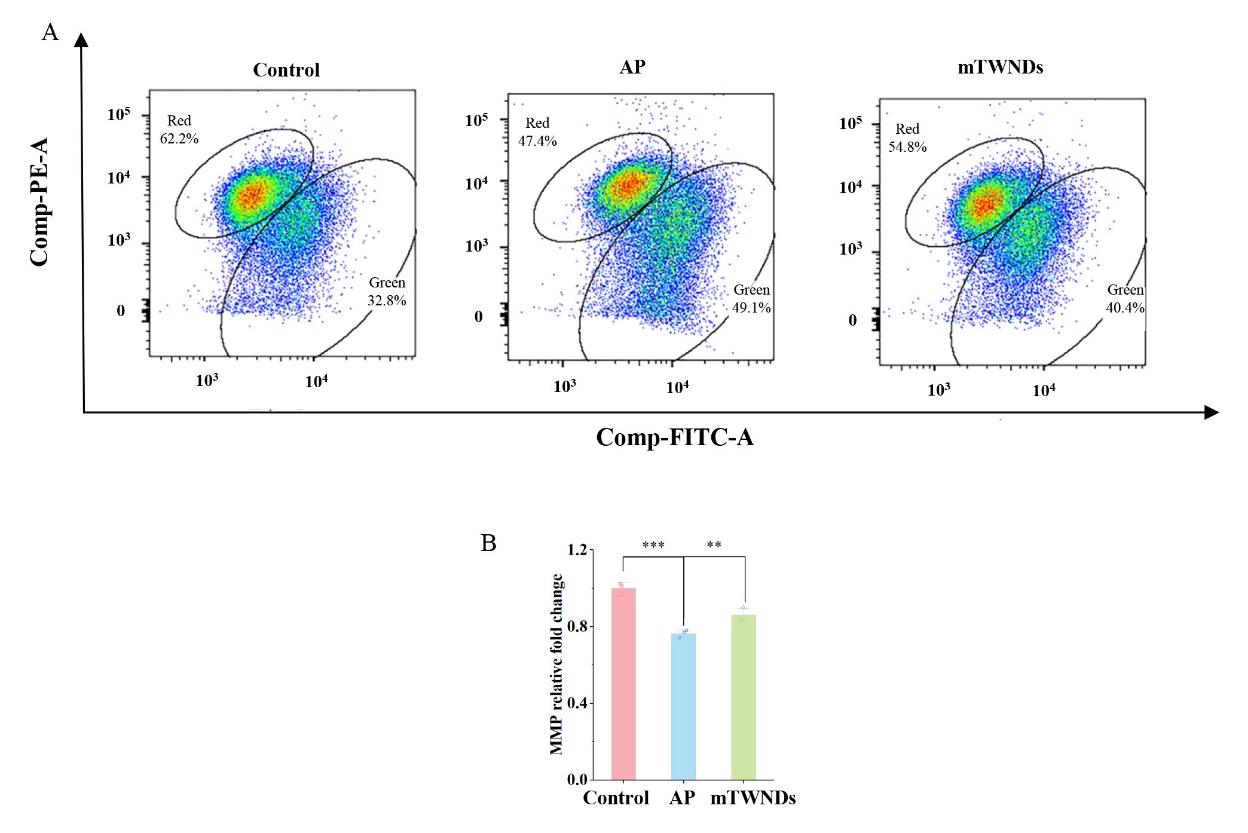


**Figure S33.** Detection (A) and quantification (B) of JC-1 flow cytometry results of PPACs under different treatments. Data represent mean ± S.D. Statistical signiﬁcance was performed by the one-way ANOVA. (n = 3 independent experiments, ^*^ for *P* < 0.05, ^**^ for *P* < 0.01, ^***^ for *P* < 0.001, ^****^ for *P* < 0.0001 vs. AP group).


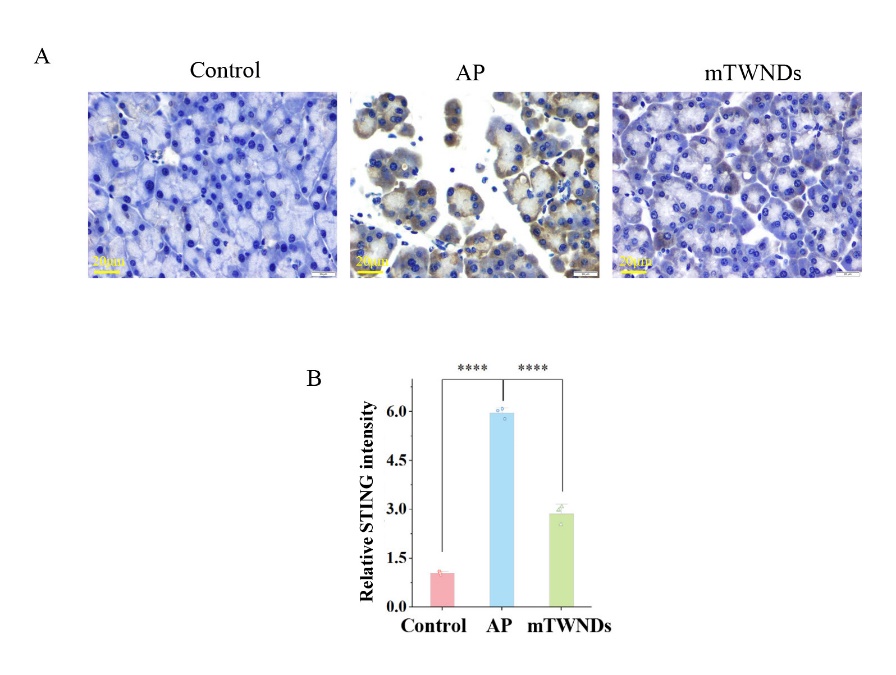


**Figure S34.** Immunohistochemical staining (A) and quantification (B) of STING in pancreatic tissues of each group. Scale bar: 20 μm. Data represent mean ± S.D. Statistical signiﬁcance was performed by the one-way ANOVA. (n = 3 animals per group, ^*^ for *P* < 0.05, ^**^ for *P* < 0.01, ^***^ for *P* < 0.001, ^****^ for *P* < 0.0001 vs. AP group).

**
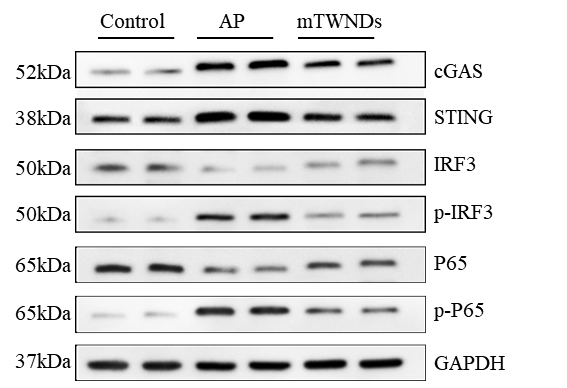
**

**Figure S35.** Western blot analysis of cGAS, STING, IRF3, p-IRF3, P65, and p-P65 proteins in pancreatic tissue homogenates from each group.


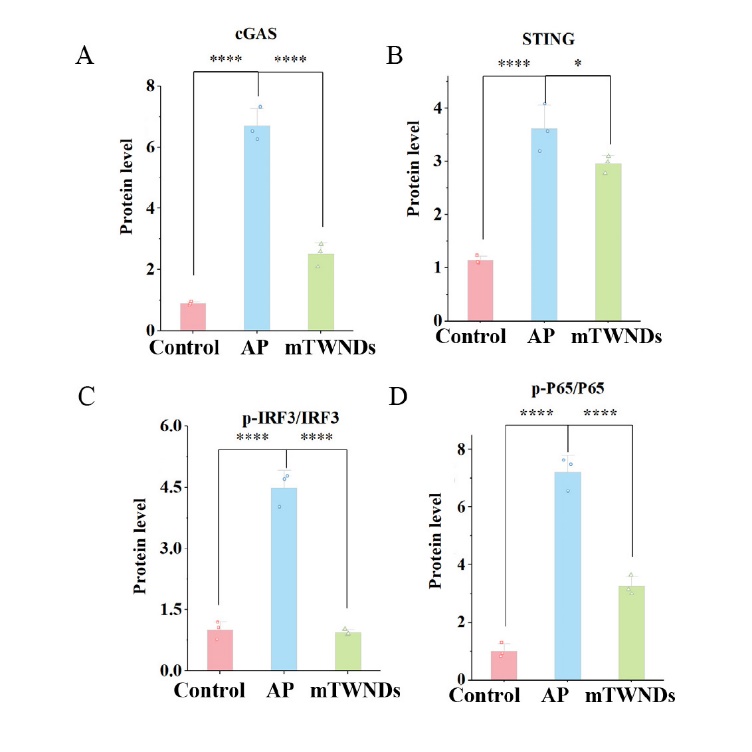


**Figure S36.** Quantification of the protein immunoblots of cGAS, STING, IRF3, p-IRF3, P65, and p-P65 in pancreatic tissue homogenates from each group. Data represent mean ± S.D. Statistical signiﬁcance was performed by the one-way ANOVA. (n = 3 animals per group, ^*^ for *P* < 0.05, ^**^ for *P* < 0.01, ^***^ for *P* < 0.001, ^****^ for *P* < 0.0001 vs. AP group).





**Figure S37.** F4/80 staining quantification in pancreatic tissues of each group. Data represent mean ± S.D. Statistical signiﬁcance was performed by the one-way ANOVA. (n = 3 animals per group, ^*^ for *P* < 0.05, ^**^ for *P* < 0.01, ^***^ for *P* < 0.001, ^****^ for *P* < 0.0001 vs. AP group).

**
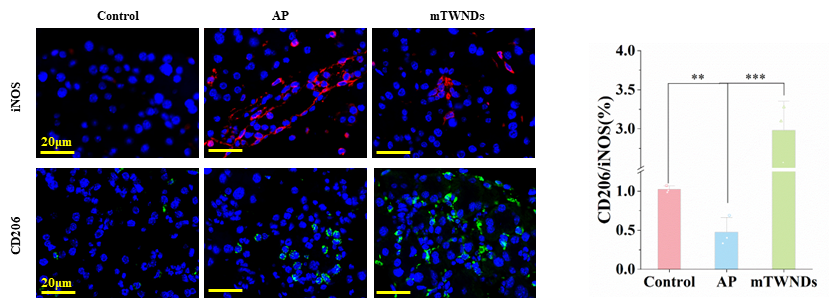
**
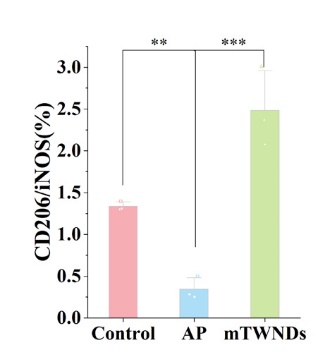


**Figure S38**. Immunofluorescence analysis of M1 (iNOS) and M2 (CD206) polarization in various groups of tissues. Data represent mean ± S.D. Statistical signiﬁcance was performed by the one-way ANOVA. (n = 3 animals per group, ^**^ for *P* < 0.01, ^***^ for *P* < 0.001 vs. AP group).


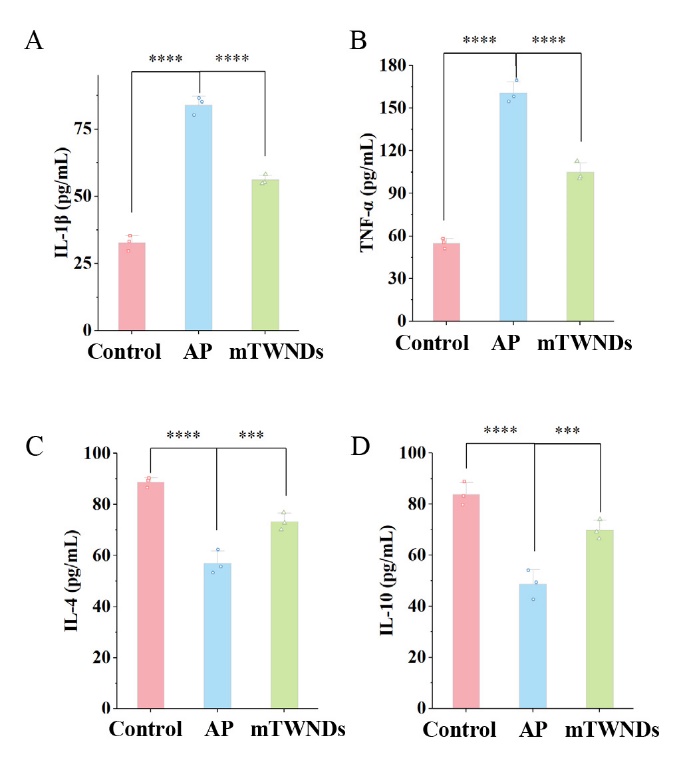


**Figure S39**. IL-1β, TNF-α, IL-4 and IL-10 levels measured in pancreatic tissues from each group. Data represent mean ± S.D. Statistical signiﬁcance was performed by the one-way ANOVA. (n = 3 animals per group, ^*^ for *P* < 0.05, ^**^ for *P* < 0.01, ^***^ for *P* < 0.001, ^****^ for *P* < 0.0001 vs. AP group).


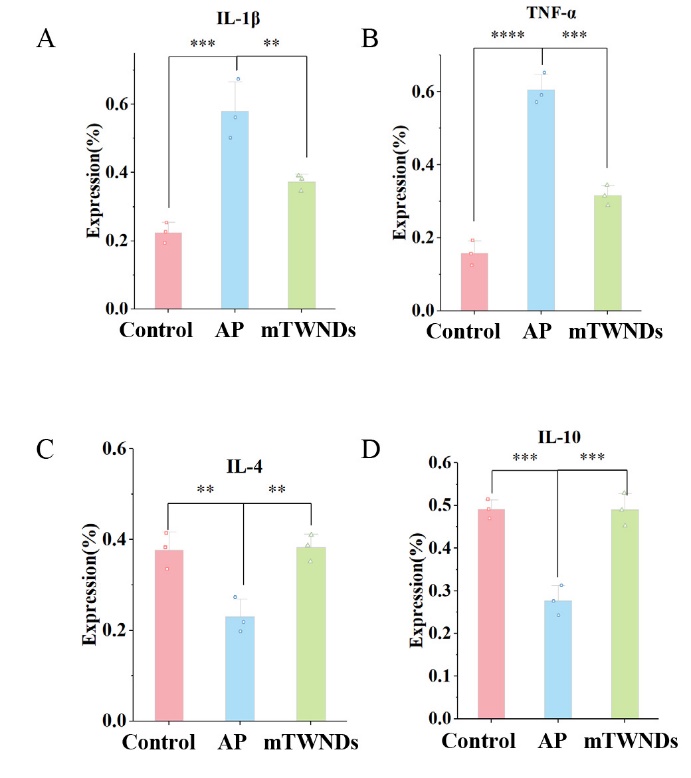


**Figure S40.** Quantitative analysis of IL-1β, TNF-α, IL-4, and IL-10 expression in immunohistochemical staining for each group. Data represent mean ± S.D. Statistical signiﬁcance was performed by the one-way ANOVA. (n = 3 animals per group, ^*^ for *P* < 0.05, ^**^ for *P* < 0.01, ^***^ for *P* < 0.001, ^****^ for *P* < 0.0001 vs. AP group).


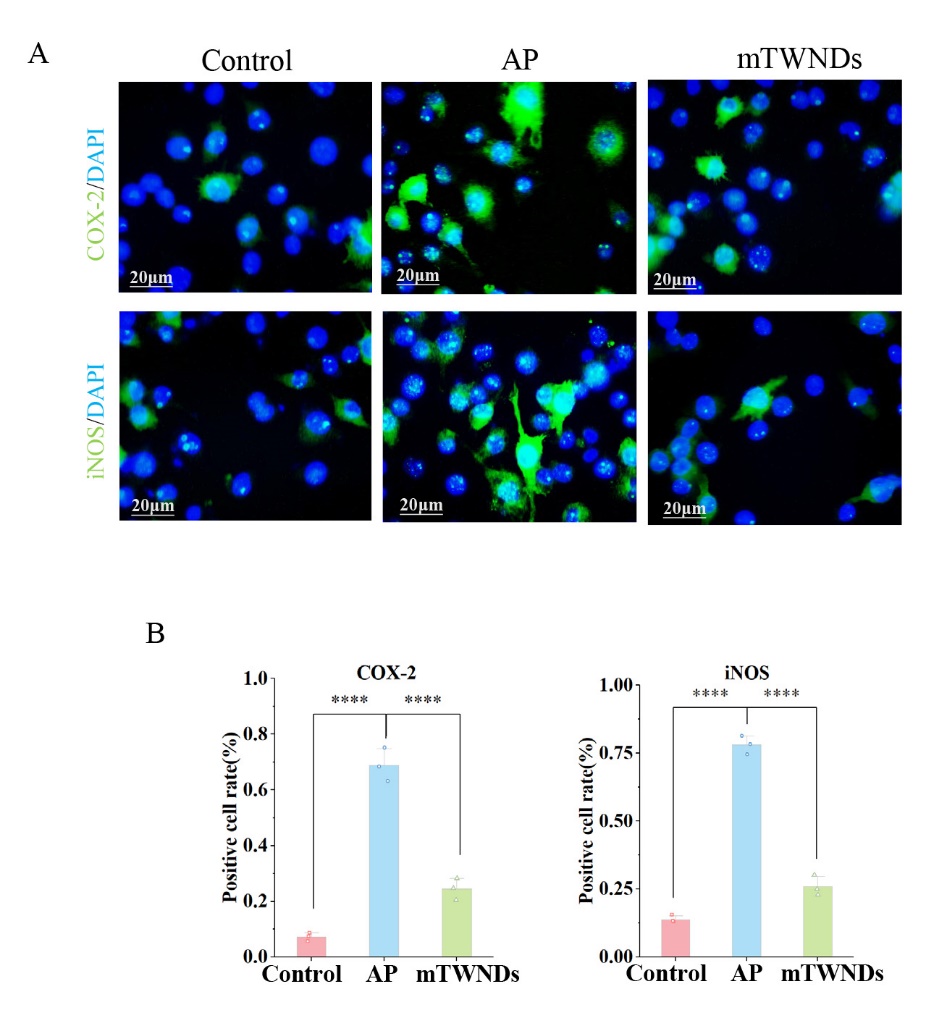


**Figure S41.** A. Immunofluorescence staining of COX-2 and iNOS in macrophages for each group. Scale bar: 20 μm. B. Quantification of COX-2-positive cells and iNOS-positive cells in (A). Data represent mean ± S.D. Statistical signiﬁcance was performed by the one-way ANOVA. (n = 3 independent experiments, ^****^ for *P* < 0.0001 vs. AP group).


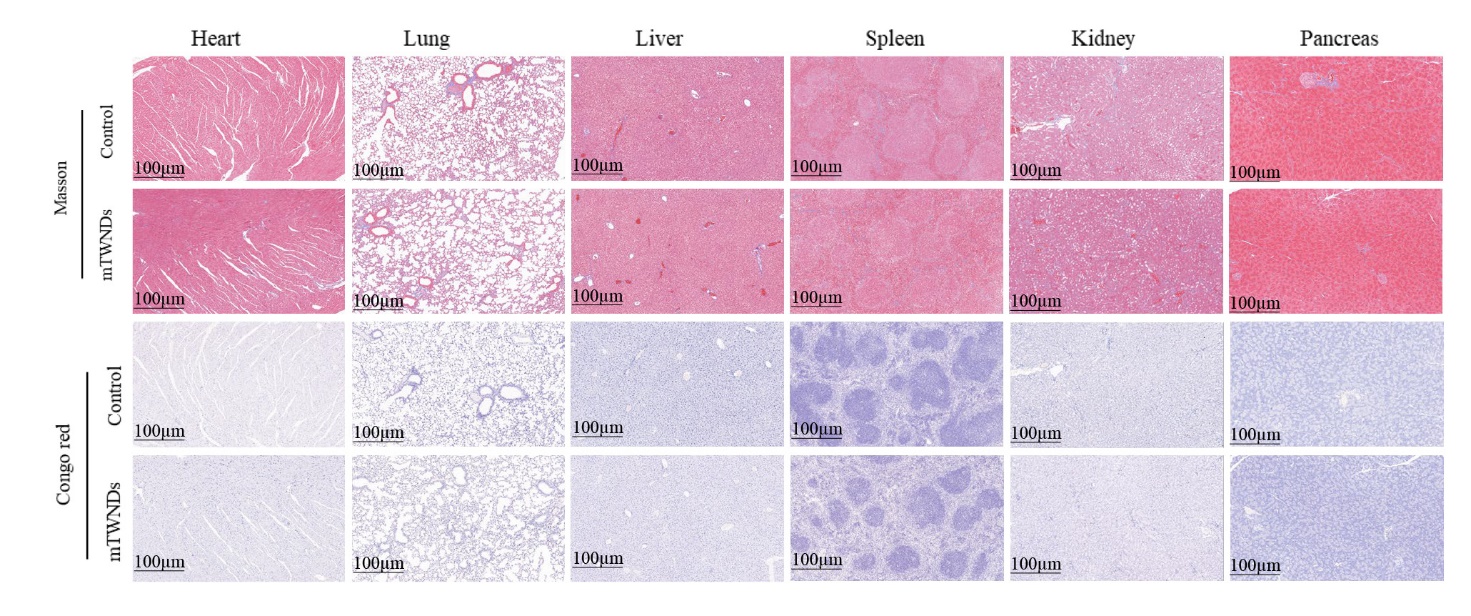


**Figure S42.** Masson staining and Congo red staining of major organs (heart, lung, liver, spleen, kidney, pancreas) of normal mice after injection with mTWNDs (20 mg/kg) for 1 day. Scale bar: 100 µm.


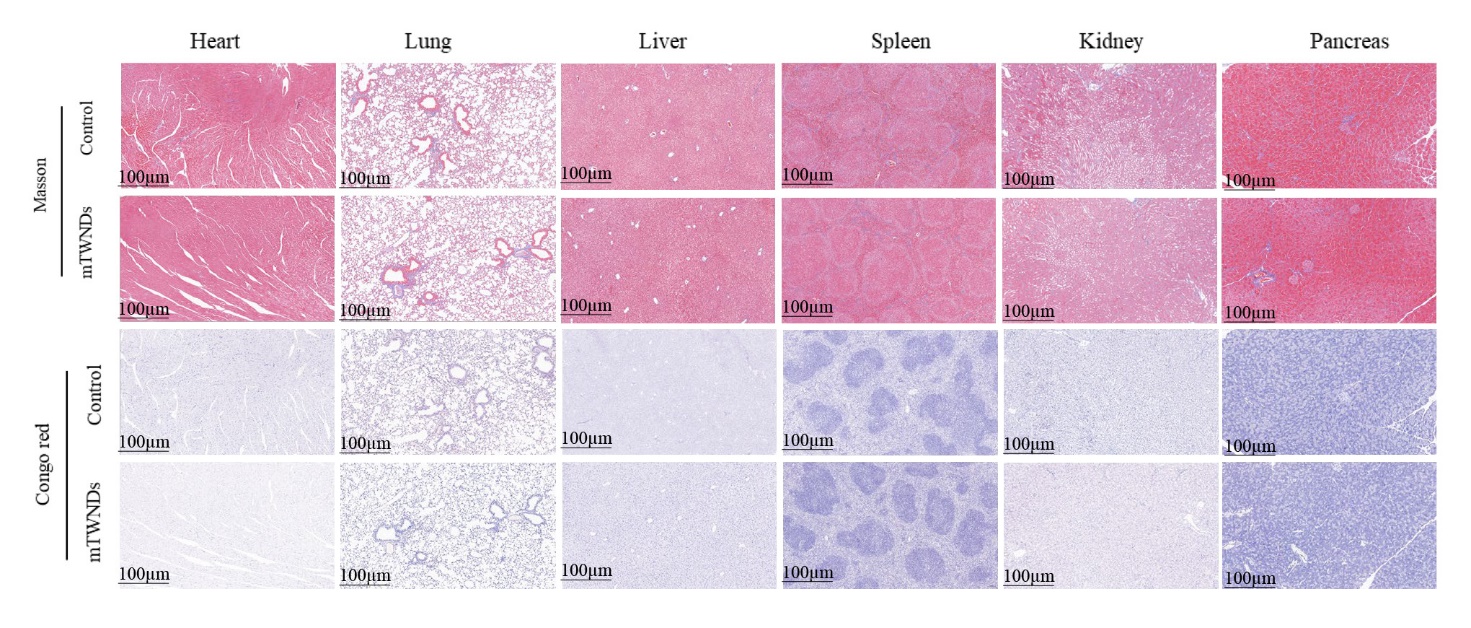


**Figure S43.** Masson staining and Congo red staining of major organs (heart, lung, liver, spleen, kidney, pancreas) of normal mice after injection with mTWNDs (20 mg/kg) for 28 days. Scale bar: 100 µm.
